# Discovery of Novel Leaf Rust Responsive microRNAs in Wheat and Prediction of Their Target Genes

**DOI:** 10.1155/2014/570176

**Published:** 2014-08-12

**Authors:** Dhananjay Kumar, Dharmendra Singh, Pulkit Kanodia, Kumble Vinod Prabhu, Manish Kumar, Kunal Mukhopadhyay

**Affiliations:** ^1^Department of Biotechnology, Birla Institute of Technology, Mesra, Ranchi 835215, India; ^2^Department of Biotechnology, Eternal University, Baru Sahib, Himachal Pradesh 173101, India; ^3^National Phytotron Facility, IARI, New Delhi 110012, India

## Abstract

MicroRNAs are endogenous small noncoding RNAs which play critical roles in gene regulation. Few wheat (*Triticum aestivum* L.) miRNA sequences are available in miRBase repertoire and knowledge of their biological functions related to biotic stress is limited. We identified 52 miRNAs, belonging to 19 families, from next-generation transcriptome sequence data based on homology search. One wheat specific novel miRNA was identified but could not be ascribed or assigned to any known miRNA family. Differentially expressed 22 miRNAs were found between susceptible and resistant wheat near-isogenic lines inoculated with leaf rust pathogen *Puccinia triticina* and compared with mock inoculated controls. Most miRNAs were more upregulated in susceptible NIL compared to resistant NIL. We identified 1306 potential target genes for these 52 miRNAs with vital roles in response to stimuli, signaling, and diverse metabolic and cellular processes. Gene ontology analysis showed 66, 20, and 35 target genes to be categorized into biological process, molecular function, and cellular component, respectively. A miRNA-mediated regulatory network revealed relationships among the components of the targetome. The present study provides insight into potential miRNAs with probable roles in leaf rust pathogenesis and their target genes in wheat which establish a foundation for future studies.

## 1. Introduction

Bread wheat (*Triticum aestivum* L.) is the second most important food crop after rice based on international commodity price and third in order of production after maize and rice. Wheat is extensively grown throughout the world and provides protein content, as well as 20% of basic caloric value to humans [1]. Wheat is the last major cereal crop for which complete genome sequence is not available due to its large genome size (16.9 GB), high proportion of transposable and repetitive DNA element, and presence of three closely related genomes (AABBDD, 2n = 6x = 42) [2]. However, recent technological advances such as next-generation sequencing platforms now offer large scale programs that delivered 5x genomic sequence resources for wheat [3]. Leaf rust is a major disease of wheat caused by the obligate biotrophic fungus* Puccinia triticina *Eriks that results in 10% yield loss annually [4]. More than 60 leaf rust resistant (*Lr*) genes had been identified from wheat and its wild relatives that confer resistance in a gene-for-gene manner. Many of them had been introgressed into wheat, but due to continuous evolution of the pathogen genome resistance breaks down quickly [5]. The molecular basis underlying leaf rust-wheat interaction is still not completely understood and as of date is a major focus of research.

Small RNAs, broadly divided into microRNAs (miRNA) and small interfering RNAs (siRNA), are global regulators of gene expression mainly through posttranscriptional or occasionally by translational repression in plants [6–8]. They are noncoding, single-stranded RNAs of 18–24 nt in length and are characterized by stem-loop structures of their precursors [9]. MiRNA genes are located mostly within noncoding regions of genomes and usually transcribed from promoters by RNA polymerase II [10, 11]. MiRNA genes represent about 1-2% of the known eukaryotic genomes and constitute an important class of fine-tuning regulators that reprogram numerous transcription events implicated in several physiological or disease-associated cellular processes by interacting with each other in regulatory networks [12, 13]. Studies in plants have revealed their key roles in diverse regulatory pathways, including auxin signaling, meristem boundary formation, organ separation, leaf development and polarity, lateral root formation, transition from juvenile to adult vegetative phase and from vegetative to flowering phase, floral organ identity and reproduction, and defense response against different biotic and abiotic stress [14, 15]. A total of 153 miRNAs were identified in wheat, of which 24 and 12 miRNAs were responsive to powdery mildew infection and heat stress, respectively [16]. The authors using* in silico *techniques also predicted 149 target genes which are potentially regulated by those miRNAs. In rice, a “osa-miR7695” miRNA through natural selection and domestication events during evolution negatively regulates an alternatively spliced transcript of natural resistance-associated macrophage protein 6 (OsNramp6) [14]. Overexpression of the miRNA in rice also conferred blast fungal pathogen resistance. This study highlights a miRNA-mediated regulation of OsNramp6 in disease resistance, whilst illustrating the existence of a novel regulatory network that integrates miRNA function and mRNA processing in relation to plant immunity.

Considering the importance of miRNAs in gene regulation, two major categories of approaches have been applied for their investigation [17]. Besides the labor-intensive experimental approaches, comparative genomics-based computational strategies are faster, affordable, and effective and are the major contributors to miRBase repertoire [17]. Since most miRNAs are evolutionary and highly conserved, comparative genomics-based prediction of new miRNA homologous from previously known miRNA sequences is a powerful approach [18]. Different computational miRNA finding strategies have been developed based on the core principle of searching for conserved sequences between different species that can fold into extended hairpins [19]. The biogenesis of miRNAs suggests that it is possible to mine miRNAs and elucidate their functions by searching repository of available expressed sequence tags (ESTs) with known miRNAs in absence of a sequenced genome [18, 20–22]. Currently, several computational approaches have been used to successfully identify potential miRNA targets in mRNA sequences or to select potential targets for experimental validation [23].

Next-generation sequencing technologies provide a rapid and high-throughput approach to enable discovery of novel species-specific or low-abundance miRNAs. Since its first use in the model species* Arabidopsis* [24], high-throughput sequencing technologies have been successfully applied in many plant species, such as rice [25], poplar [26], grapevine [27],* Medicago truncatula* [28],* Taxus chinensis* [29], foxtail millet [30], wheat, and* Brachypodium* [31] for identification of miRNAs. A total of 58 wheat miRNAs were identified by 454 sequencing, [32] and 81 novel miRNAs were identified through Solexa high-throughput sequencing [16]. In a recent study, numerous miRNAs were predicted on chromosome 6B of wheat; many of them located on repeat regions, DNA transposons, and retrotransposons suggesting propagation of miRNA genes in the wheat genome [33]. Although wheat is one of the most cultivated crops in the world, limited researches have been performed on wheat miRNAs and no studies have been reported on roles of miRNAs during wheat-leaf rust infection. In this study, new miRNAs were mined from the next-generation transcriptome sequencing data prepared from mock and pathogen inoculated wheat near-isogenic lines (NILs) for the purpose of understanding their roles in rust disease development, metabolism, and other associated physiological processes.

## 2. Materials and Methods

### 2.1. Plant Material, Pathogen, and Inoculation Treatments

Two wheat lines, HD2329, a seedling leaf rust susceptible phenotype, and its NIL HD2329+*Lr*28, a seedling leaf rust resistant with nest-immune (0-0;) phenotype, were selected for the study. The* Lr28* gene was derived from* Aegilops speltoides *(Tausch) that is effective against all pathotypes of the pathogen in India [34].* Puccinia triticina* pathotype 77-5, the most predominant and devastating pathotype in all parts of the Indian subcontinent, was selected as experimental pathogen. Plant growth, pathogen inoculum preparation, inoculation of the pathogen, and infectivity screening were performed at the National Phytotron Facility, IARI, New Delhi, exactly as mentioned in Singh et al. [35].

### 2.2. RNA Isolation, SAGE Library Preparation, and SOLiD

Leaf tissues were pooled from 15 seedlings; each mock and pathogen inoculated NILs at 24 hours after inoculation (hpi) and quickly dipped in liquid nitrogen. Total RNA was isolated using TRI Reagent (Molecular Research Center Inc., USA) as recommended by manufacturer. The RNA isolation time-point was based on earlier studies on development of infection structures [36] and activation of resistant signaling genes [35, 37]. The integrity of the isolated RNAs was confirmed on an Agilent Bioanalyser 2100. Four serial analysis of gene expression (SAGE) libraries were prepared from the isolated RNAs [coded as (i) S-M: HD2329 mock inoculated, (ii) S-PI: HD2329 pathogen inoculated, (iii) R-M: HD2329+*Lr*28 mock inoculated, and (iv) R-PI: HD2329+*Lr*28 pathogen inoculated] using SOLiD SAGE kit (Applied Biosystems, CA, USA) following the recommended protocol and sequenced using Sequencing by Oligonucleotide Ligation and Detection (SOLiD) technique commercially. The sequences had been submitted to NCBI SRA061917 (BioSample accession as SAMM01820702, SAMM01820703, SAMM01820704, and SAMM01820705).

### 2.3. Prediction of Novel and Conserved miRNA in Wheat

**Figure 1:**
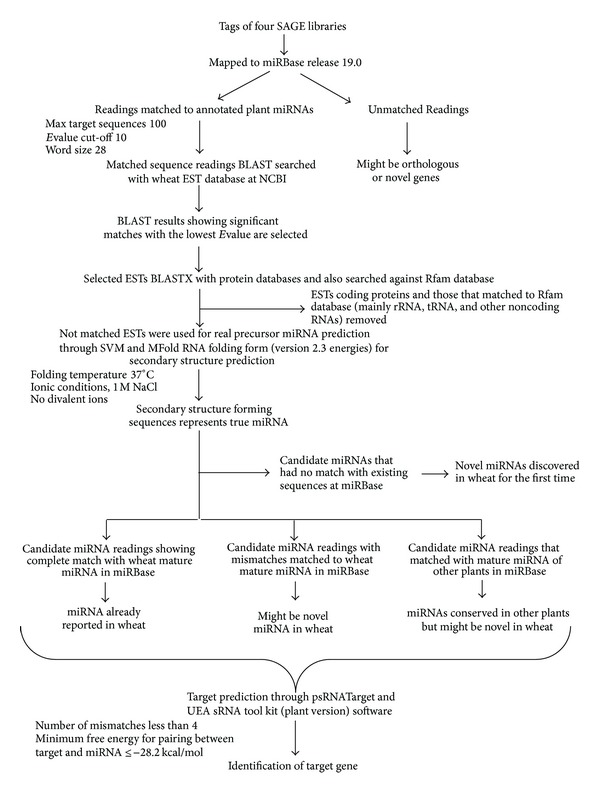
Schematic presentation of the pipeline developed to discover miRNA from NGS data and target prediction of miRNAs. Sequences of miRNAs for* Triticum aestivum, Triticum turgidum, Aegilops tauschii, Brachypodium distachyon, Hordeum vulgare, Zea mays, Oryza sativa, Saccharum *ssp.,* Sorghum bicolor, Brassica *ssp., * Arabidopsis thaliana, Glycine max*,* Vitis vinifera,* and* Solanum lycopersicum *were used for homology searches in miRBase.

After removing adaptor and redundant and ambiguous sequences, readings smaller than <18 and >28 nucleotides (nt) were discarded using small RNA analysis tool (CLC genomics workbench) [38]. Novel and conserved miRNAs were identified in wheat by annotating SAGE tags from the four libraries (S-M, S-PI, R-M, and R-PI) to existing miRNA sequences of* Triticum aestivum, Triticum turgidum, Aegilops tauschii, Brachypodium distachyon, Hordeum vulgare, Zea mays, Oryza sativa, Saccharum *ssp.,* Sorghum bicolor, Brassica *ssp.,* Arabidopsis thaliana, Glycine max*,* Vitis vinifera,* and* Solanum lycopersicum* available at miRBase (release 19, August 2012) [39, 40]. A pipeline mentioning the strategies and software used to identify and confirm the miRNAs was developed following Yao et al. [41] and Sunkar et al. [25] (Figure 1). The number of times each miRNA is represented in a particular SAGE library served as an index for estimation of their relative abundance (counts).

The initial BLASTN search with annotated miRNAs to wheat ESTs was carried out. To increase the potential of identification of miRNAs, more stringent parameters were used as follows: (1) the default word-match size was set at seven, the smallest setting that can be used for the online BLASTN program between the miRNA query and the EST sequences; (2) the expected values were set at 1,000 to increase the hit chance for more potential sequences; and (3) the sequence number of the BLAST search and the sequence alignments was set to 1,000. MiRNA sequences matching at least 18 nt and mismatches less than 3 nt were selected for further analysis. The protein coding ESTs sequences were removed with the help of BLASTX program; and only noncoding sequences were retained. BLASTN search was performed against Rfam 11.0 (http://rfam.sanger.ac.uk/) to distinguish between miRNA and other small RNA families such as rRNA, snRNA, and tRNA. Nonredundant ESTs sequences were selected with the help of Perl program. Real miRNA precursors were predicted through triplet-SVM classifier program based on support vector machine [42]. This software needs additional packages like RNAfold and LibSVM. The secondary structure of pre-miRNAs was determined using MFOLD 3.2 software [43]. The following criteria were considered for screening the candidate miRNA homologs to significantly reduce false positives as proposed by Ambros et al. [44] and Meyers et al. [32]: (i) the RNA sequence folding into an appropriate stem-loop hairpin secondary structure should contain the ~18 nt mature miRNA sequence located on one arm of the hairpin structure, (ii) the predicted mature miRNAs should not have more than 6 mismatches with the miRNA∗ sequence on the opposite arm, (iii) maximum size of 3 nt was allowed for a bulge on the miRNA sequence, (iv) miRNA precursors with secondary structures should have low minimal free energy −18 Kcal mole^−1^ and minimal free energy index (MFEI) [45], (v) the A + U content of pre-miRNA within the range of 30–70% was considered, and (vi) no loop or break in miRNA sequences was allowed. The MFEI was calculated using the equation MFEI = [(MFE/length of the pre-miRNA sequence) × 100]/(G + C) % where MFE denotes the negative folding free energies (Δ*G*). The unique miRNA sequence that perfectly matched with wheat miRNAs in miRBase was considered as known miRNAs, whereas those with partial match might be novel miRNAs for wheat. On the other hand, miRNA sequences that matched with mature miRNA sequence of other plants might be conserved in those plants but could be novel in wheat. The sequences that had no match with existing wheat miRNA sequences at miRBase were considered novel for wheat [46]. We also compared the miRNAs obtained in this study with these previously reported by Yao et al. [41] and Han et al. [20].

### 2.4. Prediction of miRNA Targets Genes and Their Functions

The putative target sites of identified miRNAs were predicted using two mostly used plant miRNA-target prediction algorithms, plant target prediction tool available on psRNATarget [47] and UEA sRNA tool kit [48] (Figure 1). Binding of miRNAs to the targets with perfect or nearly perfect complementarities was considered as this influences transcript regulation. Gaps and more than 4 mismatches between mature miRNAs and their potential target mRNA were not accepted. We subjected the potential miRNA-target genes to a functional enrichment analysis using Blast2GO software (v2.5.1) [49] with the default parameters used to obtain the GO terms for each target wheat gene. The WEGO online tool [50] was used to perform a GO enrichment analysis of the miRNA targets.

## 3. Results

### 3.1. Identification and Characterization of Potential Wheat miRNAs from SAGE Libraries

After filtering the low quality sequences, adapter sequences, and ambiguous nt, the number of tags in S-M, S-PI, R-M, and R-PI libraries was 4,949,795, 4,712,304, 3,384,144, and 3,021,557, respectively, and the average length of the sequences was 28.4–29.5 nt (Table 1). This final dataset of high quality readings was used to identify the miRNAs. The initial analysis following the developed bioinformatics pipeline (Figure 1) uncovered 258, 432, 185, and 368 predictive miRNA sequences having tag counts of 6521, 49296, 2563, and 6384 in S-M, S-PI, R-M, and R-PI libraries, respectively. The large number of miRNAs identified in the present study in wheat suggests the wide existence of miRNAs in monocot species that might play important roles in biotic stress besides growth and development. Alteration in tag counts also indicated differential expression of the identified miRNAs in response to leaf rust pathogenesis. We obtained 42, 43, 30, and 27 miRNAs from S-M, S-PI, R-M, and R-PI libraries, respectively, that provided a total of 52 unique miRNAs. All these predicted 52 miRNAs successfully accredited the criteria for identification of candidate miRNA [44]. Many miRNAs could not be uncovered due to availability of limited EST sequences and lack of completely sequenced wheat genome.

**Figure 2:**
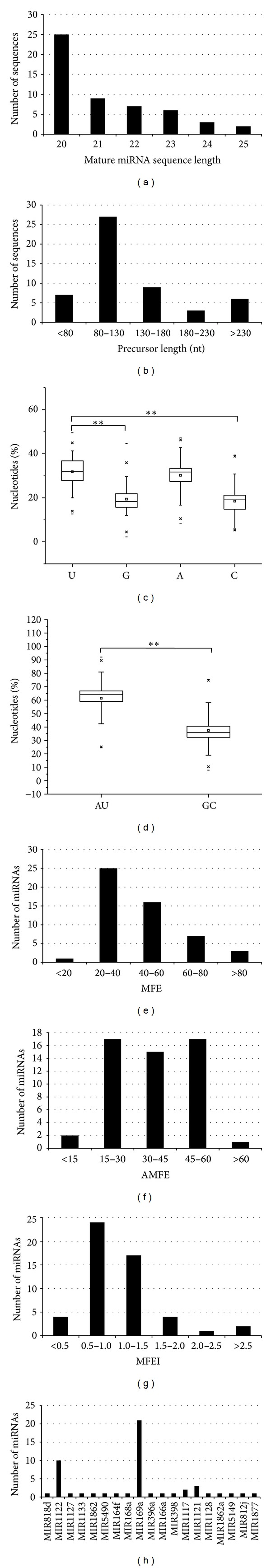
Characterization of miRNAs identified in wheat. Length distribution of mature miRNAs (a) and pre-miRNAs (b); the distribution of four nucleotides (c and d), MFE (e), AMFE (f), and MFEI (g) in the pre-miRNA sequences and miRNA families (h).

The identified mature miRNA lengths varied from 20 to 25 nt (average 21.21 ± 1.47 nt) with the 20 nt length being the predominant size (Figure 2(a)) indicating the typical size range for Dicer-derived products [51]. The pre-miRNA lengths ranged from 59 to 660 nt, with an average length of 139.76 ± 91.76 nt (Figure 2(b); Table 2). However, a large percentage of the pre-miRNAs (51.92%) were 80–130 nt and only 6 pre-miRNAs (11.53%) were more than 230 nt in length. The composition of the four nucleotides (A, G, C, and U) in pre-miRNA is an important indicator for species evolution and for the stabilization of RNA secondary structure. The percentage composition of each nucleotide was not evenly distributed in the identified wheat pre-miRNAs (Table 2). As in many previous studies the nucleotide uracil (U) is the most dominant in both mature miRNAs and pre-miRNAs in both plants and animals [52]. In this study also, we observed that the U content varied from 12.62% to 49.65% with an average of 31.83 ± 7.13% in the identified wheat pre-miRNAs (Figure 2(c)). A majority (88.46%) of pre-miRNAs contained more than 25% of the nucleotide U (Figure 2(c)), which is significantly higher than C (18.46 ± 7.03%) and G (19.38 ± 7.20%) nucleotides (*t*-test, *P* < 1*e* − 5). However, in the identified wheat pre-miRNAs, the GC content (37.39 ± 13.47%) was significantly lower than the AU content (61.40 ± 13.56%) (*t*-test, *P* < 1*e* − 5) (Table 2; Figure 2(d)). Our results were consistent with previous findings on Asiatic cotton [52] and rainbow trout [53] where more than half of the total number of nucleotides was A or U. In this study, we also determined the A/U and C/G ratio to be 0.96 and 1.00, respectively, which suggest the presence of more U and C nucleotides in precursor sequences. This criterion also differentiates miRNAs from other types of cellular RNAs such as tRNAs, rRNAs, and mRNAs [54].

The major criterion for determining the stability of a nucleic acid secondary structure is the minimal folding free energy (MFE). Usually, the lower the MFE is, the more stable the secondary structure of the RNA molecule is. The MFE of the identified wheat pre-miRNAs varied from −17.40 to −143.40 kcal mol^−1^ with an average of −44.62 ± 21.86 kcal mol^−1^ (Table 2; Figure 2(e)). The MFEs for a majority of wheat pre-miRNAs ranged from −20 to −40 kcal mol^−1^. This large variation in MFE values signifies variation in their length. For better measurement of stability of RNA secondary structures, the adjusted minimal folding free energy (AMFE) strategy was used [55]. AMFE is the MFE of a RNA/DNA sequence that is 100 nt in length. The AMFE of the identified wheat pre-miRNAs ranged from −13.63 to −62.67 kcal mol^−1^ with an average of −36.01 ± 13.66 kcal mol^−1^ (Table 2), which is a smaller range as compared with the MFE range. Most of the wheat pre-miRNAs identified in this study have AMFE values between −15 and −60 kcal mol^−1^ (Figure 2(f)). The minimal folding free energy index (MFEI), a new standard for assaying miRNAs that can distinguish miRNAs from other coding and noncoding RNAs, revealed MFEI of the identified wheat pre-miRNAs from 0.38 to 3.8 with an average of 1.07 ± 0.63 (Table 2; Figure 2(g)).

Using the BLASTN results and the secondary structure of the sequences, we identified 52 potential miRNAs from the high-throughput sequences of the SAGE libraries. Of these 52 miRNAs, 51 belonged to 19 families. However, the miRNAs identified in the present study were not evenly distributed among the families. The highest representation of 21 miRNAs in family MIR169a and 10 miRNAs in family MIR1122 was observed (Figure 2(h); see Supplementary Table  S1 in the Supplementary Material available online at http://dx.doi.org/10.1155/2014/570176). MiRNAs belonging to the same family are derived from common ancestors and generally perform similar physiological processes. The function of MIR169a and MIR1122 families, having higher representation in the present study, contained miRNAs with defense responses to biotic and abiotic stresses. One wheat specific miRNA, TamiR18, was discovered from the dataset: but this miRNA could not be ascertained to any specific miRNA family. All other identified miRNAs have partial sequence similarity to known miRNAs from other plants but were identified in wheat for the first time (Supplementary Table S1). Therefore, these 51 miRNAs are conserved for other plants but identified for the first time in wheat. The majority (47 miRNAs) of the identified miRNAs were obtained from the plus strand. However, there were five miRNAs identified from the minus strand of certain ESTs (Supplementary Table S1). All predicted miRNA secondary structures showed 11–20 nt engaged in Watson-Crick or G/U base pairing between the mature miRNA and miRNA* located on opposite arms of the hairpin structure (Supplementary Figure S1). The mature miRNA sequences could be located within either the 3′ or the 5′ arm of the secondary stem-loop hairpin structures (Supplementary Figure S1). Among the 52 identified miRNAs, 25 were located within the 3′ arm, and the rest 27 miRNAs were located within the 5′ arm.

### 3.2. Expression Profiling of Identified miRNA

**Figure 3:**
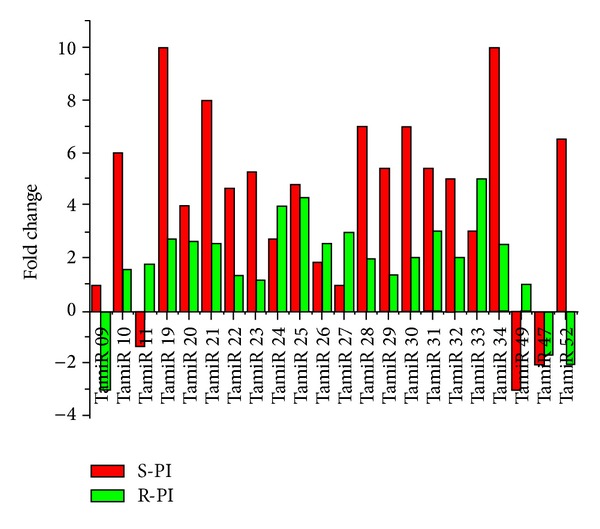
Comparative expression analysis of differentially expressed miRNAs in wheat NILs in response to leaf rust pathogen.

The tag counts of the SAGE libraries were used for expression profile analysis from mock and pathogen inoculated susceptible and resistant plants. The fold changes due to pathogen inoculation were calculated by comparing the miRNA counts in infected susceptible and resistant plants with their respective mock inoculated controls. The obtained miRNA counts suggested that most of the miRNAs were induced in S-PI and R-PI libraries in response to pathogen attack. It was observed that tag counts in S-PI library increased enormously as compared to R-PI reflecting its plausible role in suppression of disease progression in susceptible plants during leaf rust infection (Figure 3; Supplementary Table S1). On the contrary, expression of most miRNAs was comparatively low in the resistant plants after pathogenesis (Figure 3) due to the protective role of the leaf rust resistant* Lr*28 gene. We found 22 miRNAs with differential counts among the four SAGE libraries suggesting their role in basal defense and in response to leaf rust infection. Among the differentially expressed miRNAs, 17 miRNAs are upregulated and only one miRNA, TamiR47, was downregulated in both susceptible and resistant NILs due to leaf rust infection. It was observed that most miRNAs were more upregulated in the susceptible NIL. The miRNAs (TamiR09 and TamiR52) and (TamiR11 and TamiR49) were downregulated only in resistant and susceptible NILs, respectively, due to leaf rust infection. In summary, the tag-based differential expression patterns reflected a comprehensive characterization and specificities of the identified miRNAs with respect to leaf rust infection in wheat.

### 3.3. Potential miRNA-Target Prediction and Functional Analysis

**Figure 4:**
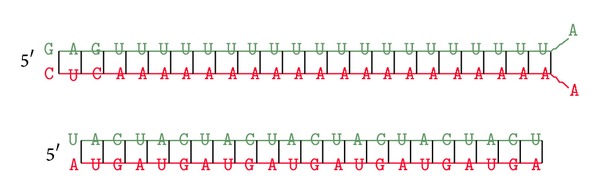
Representative miRNAs (green) hybridized to target (red) as obtained with RNAhybrid software.

MicroRNAs regulate expression of specific genes at posttranscriptional level, either by endolytic cleavage of mRNA transcripts or by translational inhibition or by both methods [56]. To better understand the biological functions, particularly the development of leaf rust diseases of the newly identified wheat miRNAs, we searched for putative target genes using the psRNATarget program with default parameters against the* Triticum aestivum* (wheat) Unigene and DFCI Gene Index (TAGI), version 12, release data 2010.04.18. We adjusted the expectation value to 2.0 to reduce the prediction of false positives [47]. A total of 1306 potential targets were identified for the 52 miRNAs based on their perfect or nearly perfect complementarities with the target sequences in wheat (Figure 4; Supplementary Table S2). The miRNA family “miR169a” showed the highest (527) numbers of independent target genes followed by “miR1122” family with 253 and miR1121 family with 72 target genes.

Plant miRNA-target sites are predominantly located within ORFs [57]. This allowed prediction of on average seven targets for every new miRNA (Supplementary Table S2). Many identified miRNAs were found to regulate genes encoding for structural proteins like Histones H1, H2A, and H3, chromosome segregation proteins, and ribosomal proteins. Numerous miRNAs targeted proteins with metabolic activities such as phenylalanine ammonia-lyase, ribulose-1,5-bisphosphate carboxylase/oxygenase, and other proteins of glycolysis, tricarboxylic acid cycle, cytochromes, photosynthesis, and electron transport including thioredoxin h-type, ubiquitins, and the cereal specific protein alpha-gliadin and starch branching enzymes. Many of the miRNA-target genes encoded for transcription factors involved in signaling and defense like Zn-finger proteins, leucine rich repeats, NB-ARC, F-box, WRKY, DREB, Myb, PHD, ER-EBPs, WD domain, major facilitator superfamily, DEAD-box, ATP dependent helicases, and transcriptional activators and regulators like TetR family. Some targets also included defense and stress responsive proteins like universal stress proteins, Pr1, cold responsive proteins, metallothioneins, several kinases, heat shock protein, beta-glucanases, prexidases, and chitinases. The identified miRNAs also targeted transporters like ABC, sugar, heavy metals, phosphate, and Na^+^/Pi (detail list is provided in Supplementary Table S2). It is interesting to note that target validations for nonconserved miRNAs in wheat had not been very successful, unlike conserved miRNA targets. It has been hypothesized that nonconserved and recently evolved miRNAs exist without actual targets [57]. It is also possible that the applied stringent criteria might have missed the prediction of several potential target genes [24]. The findings from this study will contribute to further understanding on the miRNAs function and regulatory mechanisms of miRNAs in wheat.

**Figure 5:**
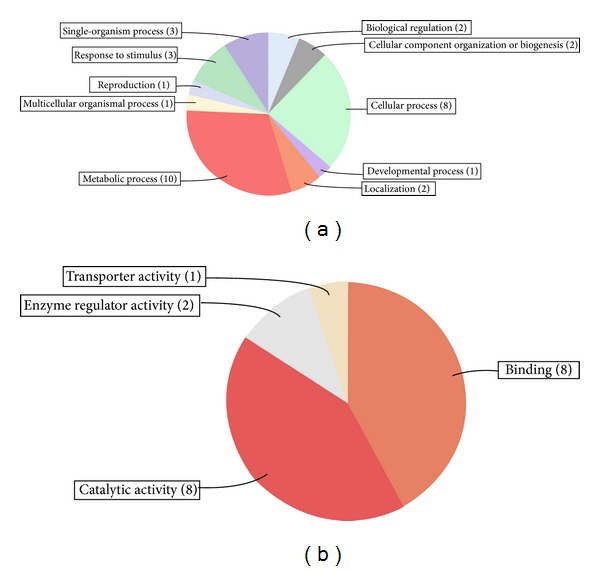
GO (gene ontology) term enrichment analysis of miRNAs target genes. Analysis of miRNAs targets within biological process (a) and molecular function (b) category.

For comprehensive annotation, all putative target transcripts were subjected to functional enrichment by gene ontology (GO) terms with the aid of Blast2GO and WEGO programs using default parameters. The identified wheat miRNA targets generated significant GO terms for further analysis. GO analysis categorized 66, 20, and 35 target genes into biological process, molecular function, and cellular component, respectively. The GO enrichment analysis showed that the predicted targets of the miRNAs were involved in a wide range of regulatory functions as well as some specific biological processes like metabolism, biosynthesis, and gene expression/transcription (Figure 5(a); Supplementary Figure S2). The transcripts representing genes with known functions were categorized by biological process and molecular function according to the ontological definitions of the GO terms. The majority of the predicted targets are involved in a broad range of biological processes such as metabolic process (10 terms), cellular process (8 terms), response to stimulus (3 terms), single-organism process (3 terms), developmental process (1 term), cellular component organization or biogenesis (2 terms), localization (2 terms), developmental process (1 term), reproduction (1 term), and biological regulation (2 terms) (Figure 5(a); Supplementary Figure S2). The putative target transcripts of miRNAs in the molecular function category were related to binding (8 terms), catalytic activity (8 terms), transporter activity (1 term), and enzyme regulator activity (2 terms) (Figure 5(b); Supplementary Figure S2). Most of the miRNA-target genes assigned to the binding category were involved in nucleic acid binding, protein binding, and ion binding. Since these sequences are encoded for transcription factors, this is in accordance with previous reports [58–60]. In addition, some of the predicted targets had annotations in the cellular component category including cell and cell parts (Supplementary Figure S2).

Detailed analysis of the predicted targets revealed that the majority of the miRNAs have more than one potential regulatory target and conversely a single target could be regulated by more than one miRNA (Supplementary Table S2). This observation leads us to focus on development of a regulatory network comprising of more than one miRNA having different targets [46]. Based on the obtained miRNA-target pairs, we constructed a molecular network using Blast2GO software (Supplementary Figure S3). The constructed complex network shows significant relationship of the targetome with GO terms related to defense response, gene expression, metabolic process, cellular biosynthetic process, response to chemical stimulus, binding (ion binding, metal ion binding, RNA binding, DNA binding, ATP binding, etc.), and catalytic activity (transferase activity and hydrolase activity) (Supplementary Figures S3A and S3B). We were also able to identify seven wheat miRNAs having target genes involved in defense and signaling in plants (Table 3).

## 4. Discussion

Numerous studies in recent years helped to discover and identify miRNAs from different plant species using next-generation sequencing approaches, followed by computational prediction methods [18, 19, 61–63]. Although numerous miRNAs have been identified in model plants like* Arabidopsis* and rice using experimental approaches, computational prediction from the EST sequences is the rapid and effective way to explore potential miRNAs in important crop plants like wheat where complete genomic sequence is still not available [17]. In the present study using comparative genomics-based search and bioinformatic tools we could identify 52 mature miRNAs in wheat belonging to 19 families. However, in addition to the highly conserved miRNAs, there are species-specific miRNAs originating from recently evolved* miRNA* genes [21, 64, 65]. These species-specific miRNAs often accumulate at lower levels and can be difficult to detect with traditional experimental based methods. The miRBase repository, as of release 19, has only 42 wheat miRNAs, a significantly inadequate number compared to other cereals (rice 708; maize 321, and* Sorghum* 242). This suggests that many more miRNAs are yet to be identified in wheat. Although the number of deeply conserved miRNA families in wheat largely remains the same as in* Arabidopsis*, all of the newly identified wheat miRNAs do not appear to be conserved in* Arabidopsis* and have predicted target genes with more diverse functions than those of conserved miRNAs.

Current literature suggests that several plant genes are involved in response to biotic and abiotic stress conditions exhibiting tissue and development stage specific expression which may be regulated at the posttranscriptional level by miRNA [41, 66, 67]. A large number of drought responsive miRNAs were identified in* Prunus persica* through high-throughput sequencing and their expression patterns were analyzed in roots and leaves [68]. At a genome-wide level 215 miRNAs and 447 miRNA targets were identified in foxtail millet and some novel foxtail millet miRNAs and their targets were validated experimentally [30]. Various pine-specific miRNA families were differentially expressed in response to infection by the rust fungus* Cronartium quercuum *f. sp.* fusiforme*, which causes fusiform rust disease in pines [69]. Some of the conserved miRNAs differentially expressed in response to powdery mildew infection in wheat such as miR156, miR159, miR164, miR171, and miR396 were downregulated whereas miR393, miR444, and miR827 were upregulated, respectively [16].

The length of majority of mature miRNAs was 20 nt as was also found in Asiatic cotton [52]. A notable aspect of this study was accretion of one novel wheat specific miRNA TamiR18. We also compared the 52 TamiRNAs identified in the present study with the 58 and 37 TamiRNAs described by Yao et al. [41] and Han et al. [20]. The search did not yield any perfectly matched miRNAs. The wheat genotypes used in the present study and those used by Yao et al. [41] were different whereas Han et al. [20] used a different pipeline targeting the miRNA registry database available at the Sanger Institute. These might be the key reasons for getting different miRNAs. Twenty-two of the identified miRNAs were differentially expressed in leaf rust susceptible and resistant NILs under mock and pathogen inoculated conditions. Many of the identified miRNAs were expressed more in susceptible plants in response to the rust pathogen in an effort to prevent the spread of the pathogen to adjacent tissues. The predicted miRNA targets included genes coding for structural proteins, metabolically active proteins, transcription factors, transcriptional activators, transcriptional regulators, and transporters, besides defense signaling and stress responsive proteins. Identification and functional characterization of entire miRNAs and their targets will lay the groundwork to unravel the complex miRNA-mediated regulatory networks involving the mysteries of host-pathogen interactions.

## 5. Conclusions

We identified 52 miRNAs that are found for the first time in wheat but conserved in other plants including one novel wheat specific miRNA. The wheat specific miRNA could not be ascertained to any known miRNA family, whereas the remaining 51 miRNAs were distributed into 19 miRNA families. Twenty-two miRNAs were differentially expressed in response to leaf rust pathogenesis. Based on the computational target prediction and GO analysis of the target genes, we found that many of these miRNAs were involved in development and biotic and abiotic stresses, thereby providing useful information regarding their regulatory roles in plant physiological processes like defense and signaling. Most putative target transcripts were involved in several biological processes related to nucleic acid binding (transcription factors) and catalytic activities. Insights into the miRNAs and their target genes will greatly contribute to the deeper understanding of posttranscriptional gene regulation in response to leaf rust induced biotic stress in wheat. The identification and characterization of miRNAs from wheat will also enrich the miRNA repertoire and provide aid in miRNA-based research on leaf rust pathogenesis in wheat.

## Supplementary Material


*Triticum aestivum* L. pre-miRNA secondary structures predicted using Mfold showing the mature miRNAs in stem portion (green). Gene Ontology (GO) categories and distribution of miRNA target genes in wheat. The results are classified in three main categories: cellular component, molecular function and biological process. The y-axis on the left indicates the percent of genes in a category, while the y-axis on the right indicates the number of genes in a specific category. Gene Ontology (GO) term enrichment analysis of the miRNAs target genes. Analysis of the targetome of miRNAs within Biological process (A) and Molecular function (B) category. The predicted regulatory relationships between miRNA targets were performed by using the online tool Blast2GO with default parameters. Wheat miRNAs identified by homolog search and secondary structure. Potential target genes for the identified miRNAs.

## Figures and Tables

**Table 1:** Statistics of small RNA sequences in all four libraries.

Library	Tag counts after removing low quality sequences	Average length of tags (nt)	Tags annotated to miRBase	Number of ESTs matched to annotated tags	Number of ESTs after BLASTX and Rfam searches	Real precursor miRNAs conformed by SVM
S-M	4,949,795	29.5	258	3253	1178	42
S-PI	4,712,304	29.3	432	4750	2293	43
R-M	3,384,144	28.6	185	1730	694	30
R-PI	3,021,557	28.4	368	4021	1298	27

**Table 2:** Statistics of the characterized parameters of wheat miRNA precursors.

Parameter	Mean	Standard deviation	Minimum	Maximum
MFE (Δ*G*, −kcal/mol)	44.62	21.86	17.4	143.4
AMFE (Δ*G*, −kcal/mol)	36.01	13.66	13.63	62.67
MFEI (Δ*G*, −kcal/mol)	1.07	0.63	0.38	3.8
Length (nt)	139.76	91.76	59	660
(G + C)%	37.39	13.47	7.9	75.58
(U + A)%	61.4	13.56	24.42	92.13
A%	30.2	7.8	8.45	47.19
C%	18.46	7.03	5.52	39.43
G%	19.38	7.2	2.22	44.65
U%	31.83	7.13	12.62	49.65
A/U ratio	0.96	0.22	0.46	1.07
C/G ratio	1	0.36	0.41	2.5

**Table 3:** Identified miRNAs having targets of pathogen secretome.

Serial number	miRNA	Target genes	Functions
1	TamiR39	Beta-glucanase	Have roles in plant defense by degrading cell walls of pathogen, thereby disrupting its deposition and contributing to pathogen death; subsequently, the released cell wall fragments act as elicitors for host defense response.
2	TamiR37, TamiR38	Peroxidase 6	Play important role in physiological processes like responses to biotic and abiotic stresses and biosynthesis of lignin and are involved in the scavenging of oxidative damage causing ROS.
3	TamiR10, TamiR43	Calreticulin like protein	Ubiquitous protein crucial for plant growth and development; powerful regulator in stress responses such as cold, drought, phytohormones, and pathogen.
4	TamiR40	Cinnamyl alcohol dehydrogenase	A key enzyme in lignin biosynthesis that catalyzes the final step in conversion of monolignols and also increases the strength and lodging resistance of stem tissues in monocot.
5	TamiR36	Pr1	Involved in plant defence responses against fungal pathogens

## Abbreviation

AMFE: Adjusted minimal folding free energy
BLAST: Basic Local Alignment Search Tool
EST: Expressed sequence tags
GO: Gene ontology
MFE: Minimal folding free energy
MFEI: Minimal folding free energy index
miRNA: microRNA
NCBI: National Center for Biotechnology Information
NIL: Near-isogenic line
Pre-miRNA: precursor miRNA
SAGE: Serial analysis of gene expression
siRNA: Small interfering RNA
SOLiD: Sequencing by Oligonucleotide Ligation and Detection
TamiRNA: * Triticum aestivum* microRNA
WEGO: web gene ontology annotation
RuBisCO: Ribulose-1,5-bisphosphate carboxylase/oxygenase.
