# Supplementary material for: Discovery of Novel Leaf Rust Responsive microRNAs in Wheat and Prediction of Their Target Genes
Source: J Nucleic Acids. 2014 Aug 12;2014:570176. doi: 10.1155/2014/570176 (PMC4144313; doi:10.1155/2014/570176)
Supplement: Supplementary file 1 — Triticum aestivum L. pre-miRNA secondary structures predicted using Mfold showing the mature miRNAs in stem portion (green). Gene Ontology (GO) categories and distribution of miRNA target genes in wheat. The results are classified in three main categories: cellular component, molecular function and biological process. The y-axis on the left indicates the percent of genes in a category, while the y-axis on the right indicates the number of genes in a specific category. Gene Ontology (GO) term enrichment analysis of the miRNAs target genes. Analysis of the targetome of miRNAs within Biological process (A) and Molecular function (B) category. The predicted regulatory relationships between miRNA targets were performed by using the online tool Blast2GO with default parameters. Wheat miRNAs identified by homolog search and secondary structure. Potential target genes for the identified miRNAs. [file 570176.f1.doc]

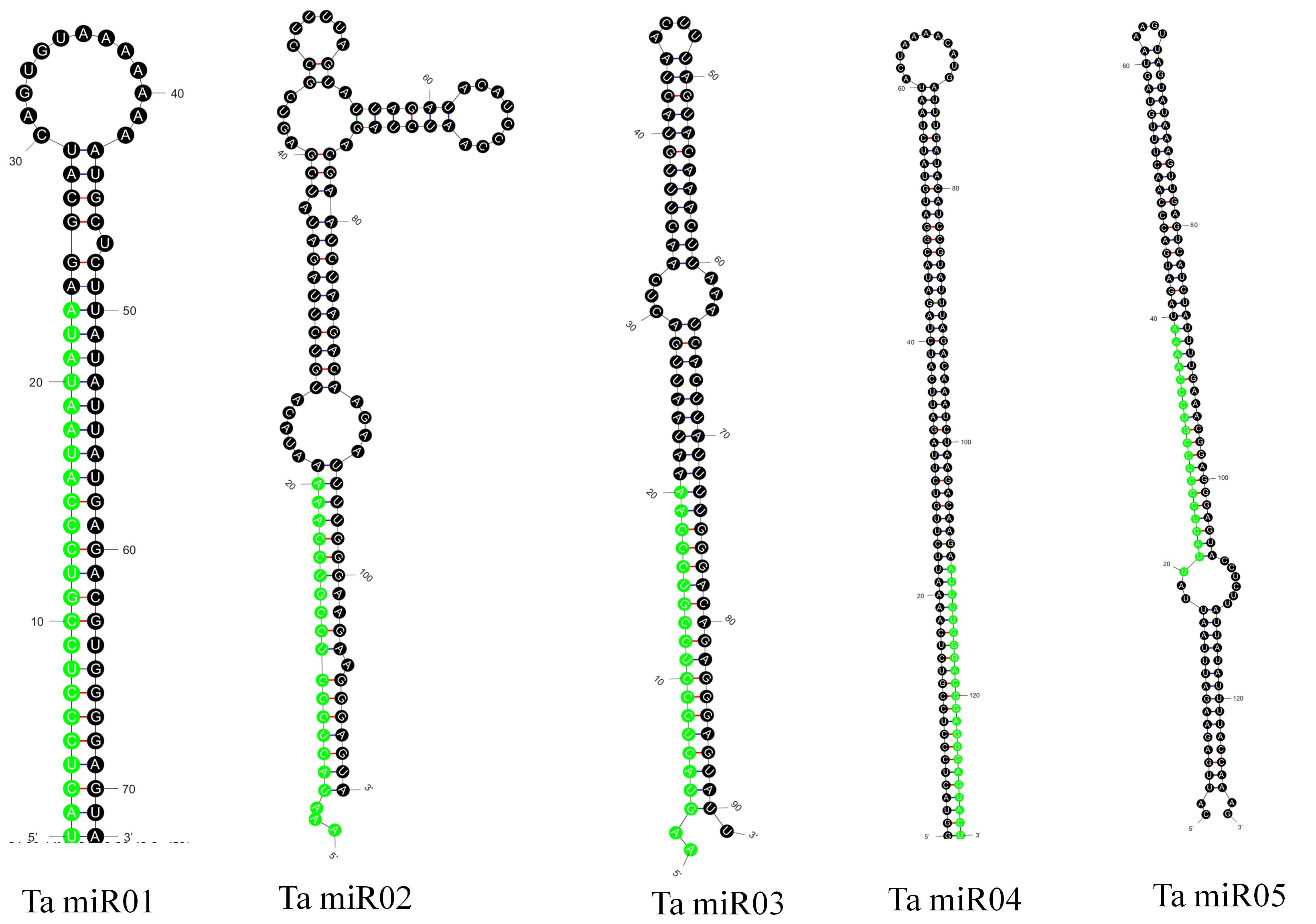


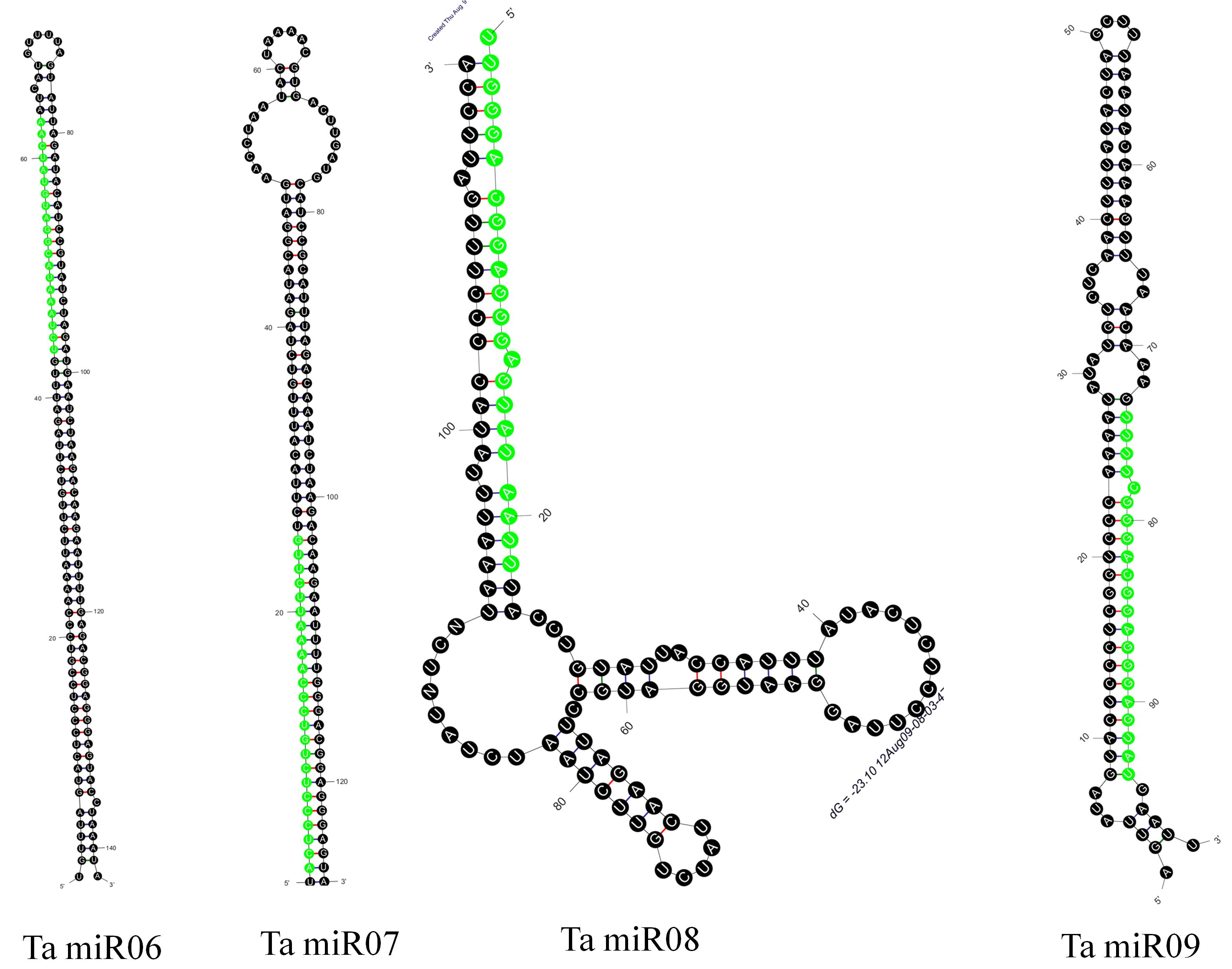


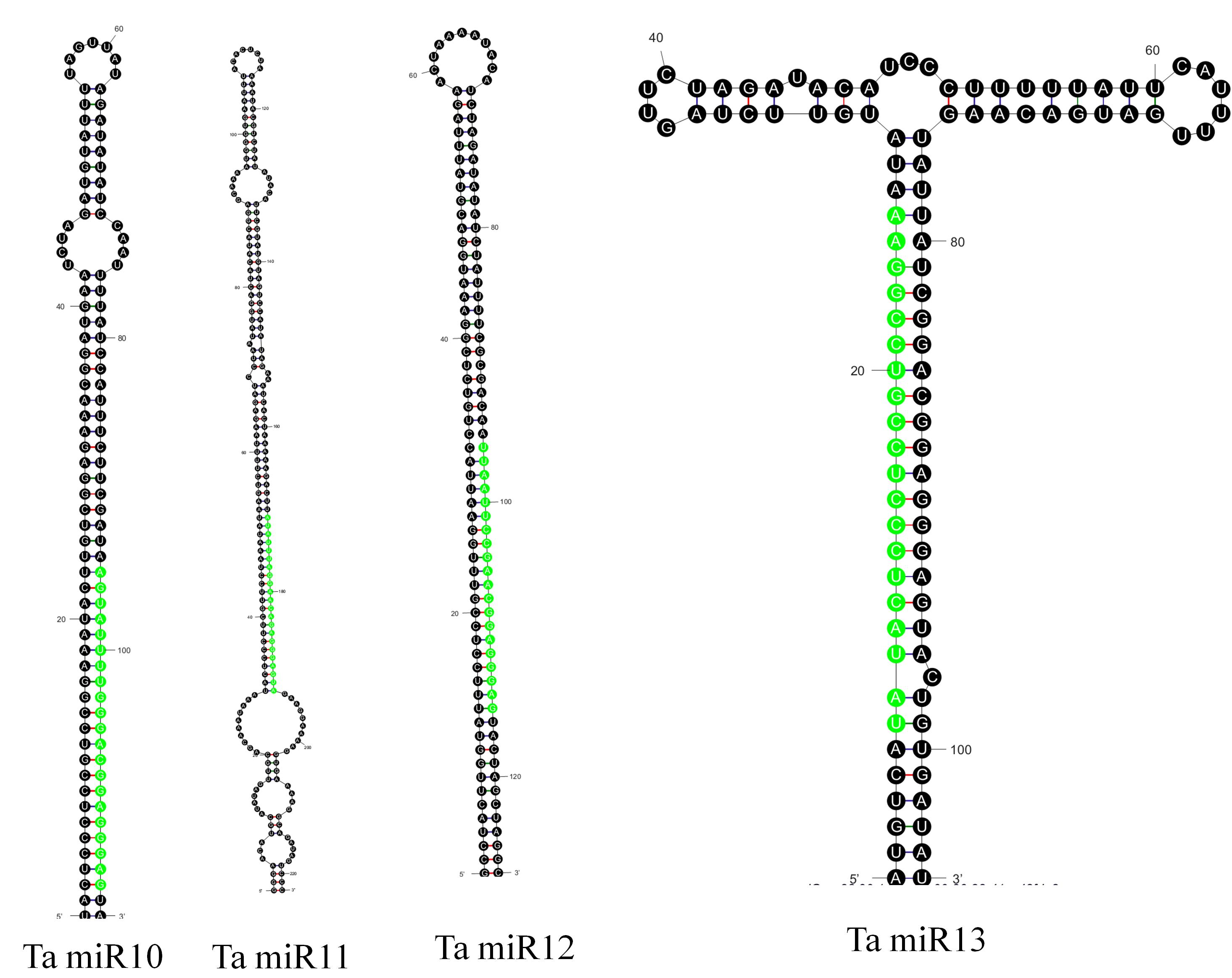


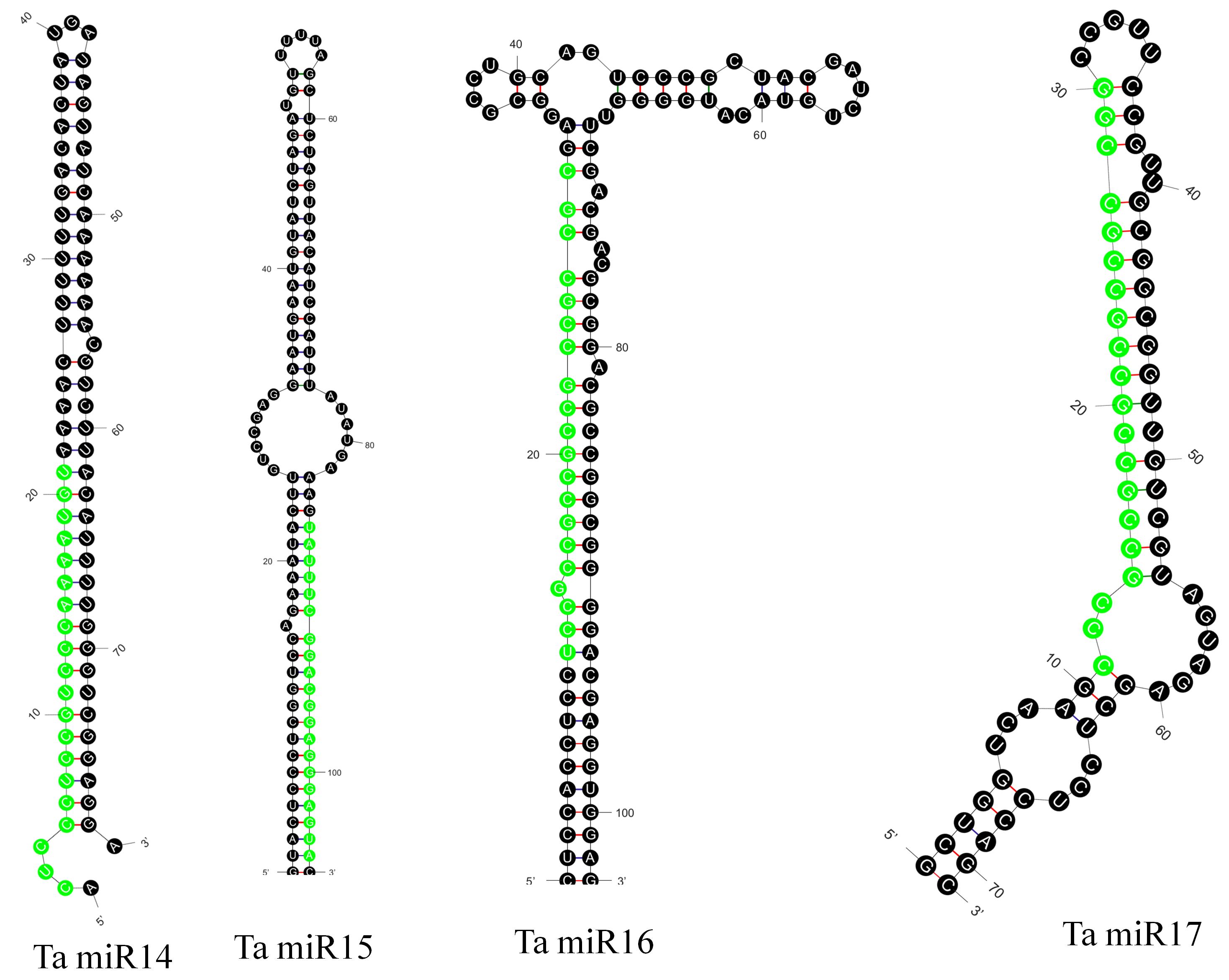


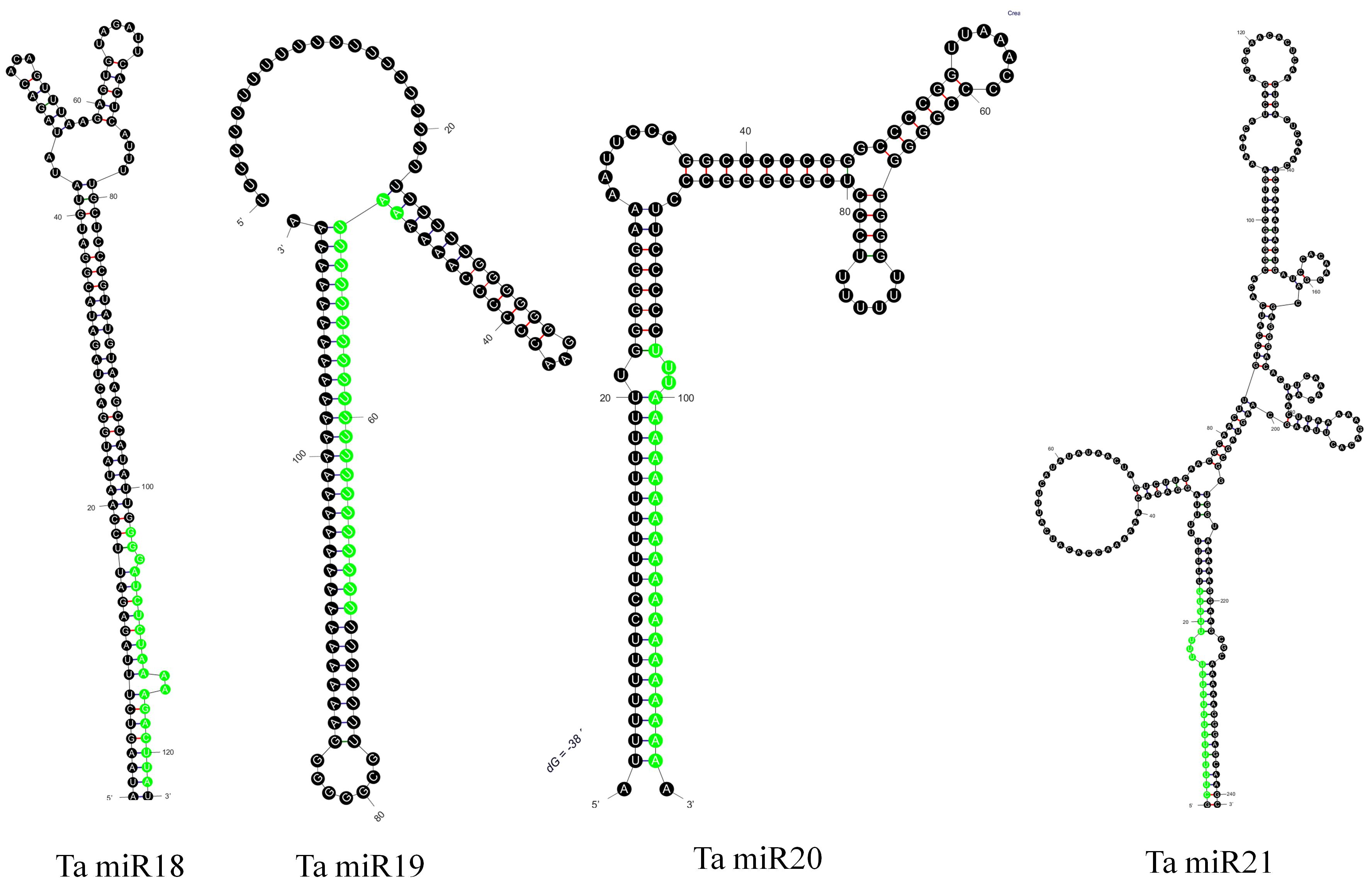


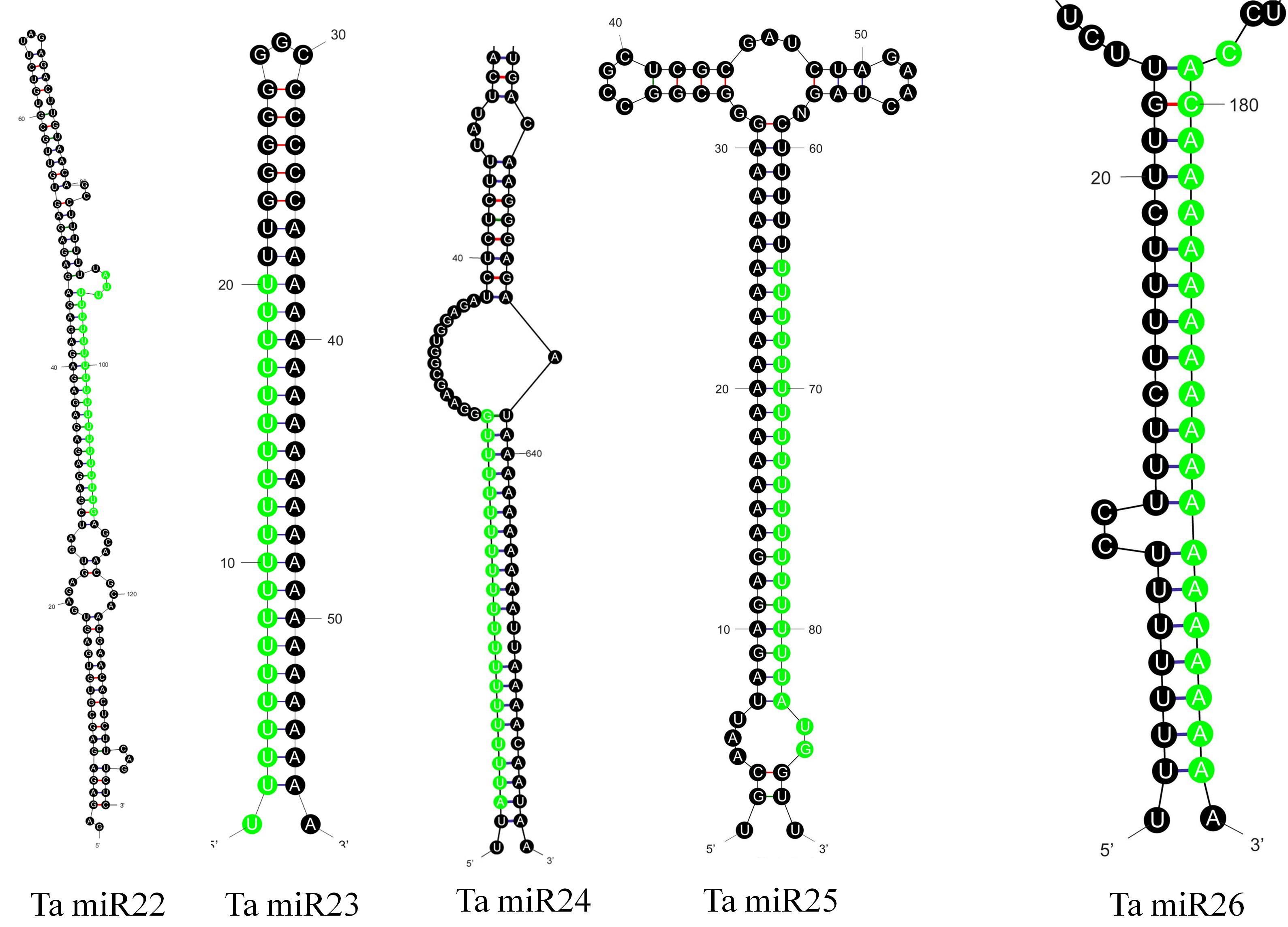


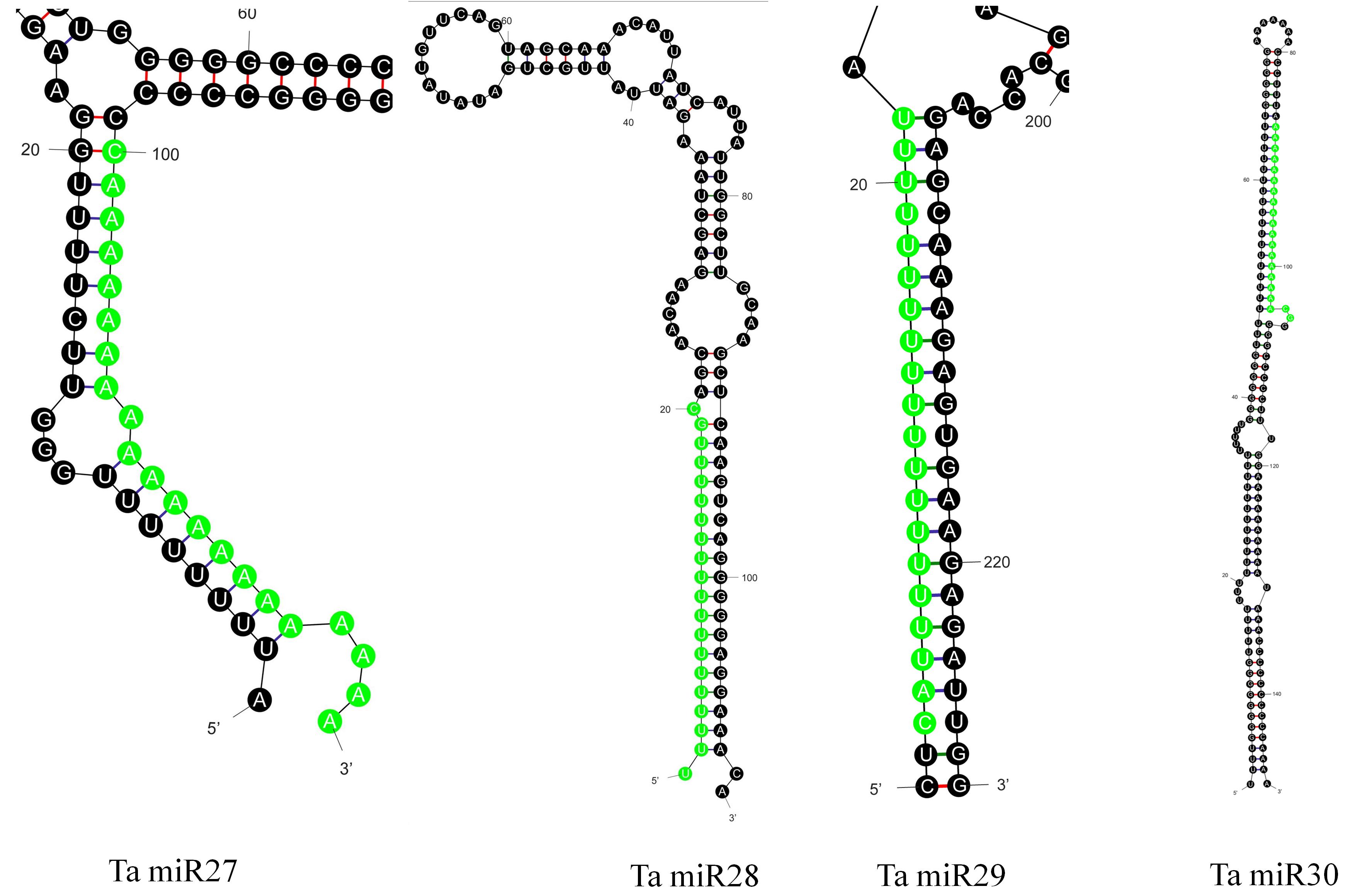


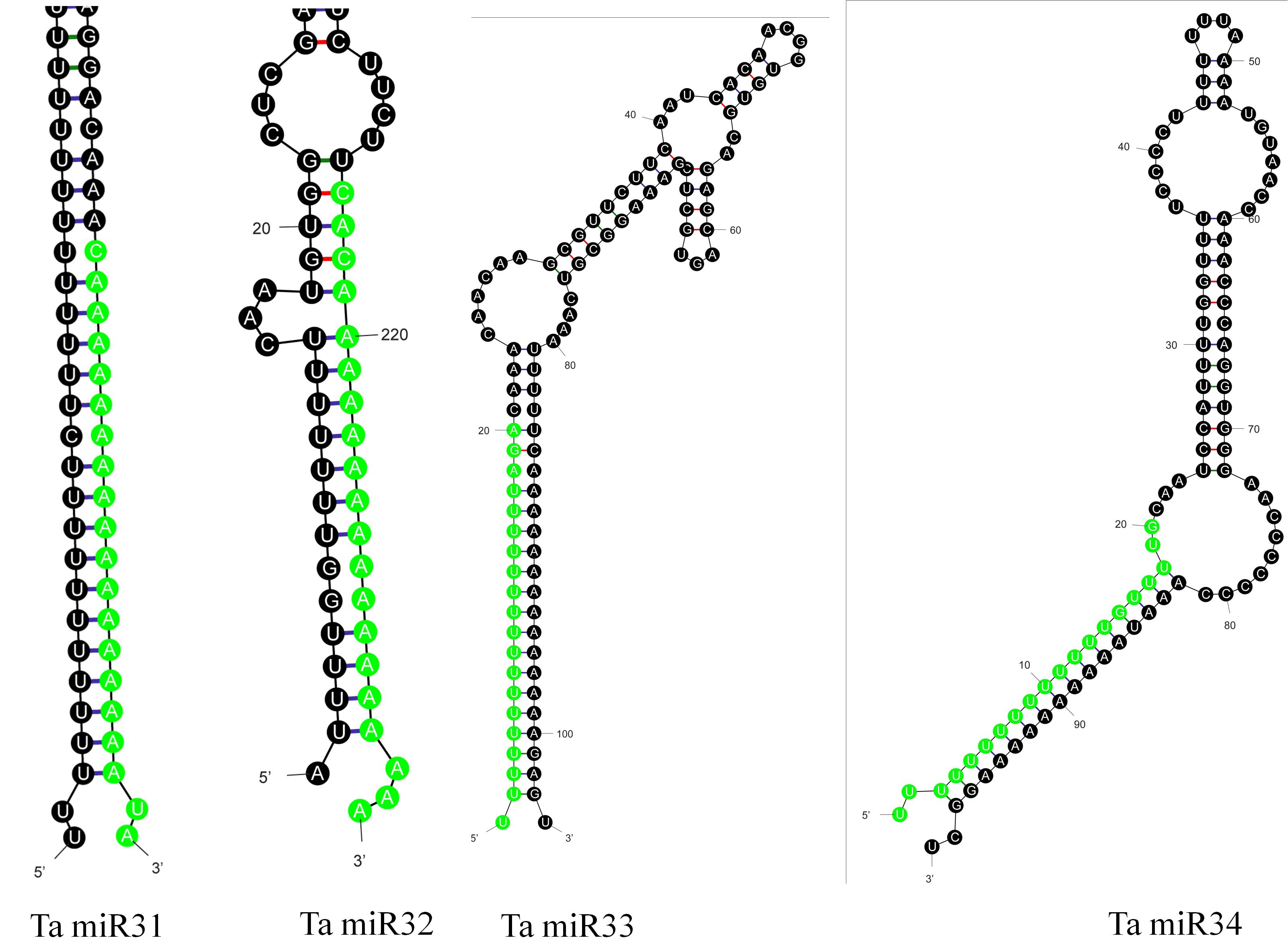


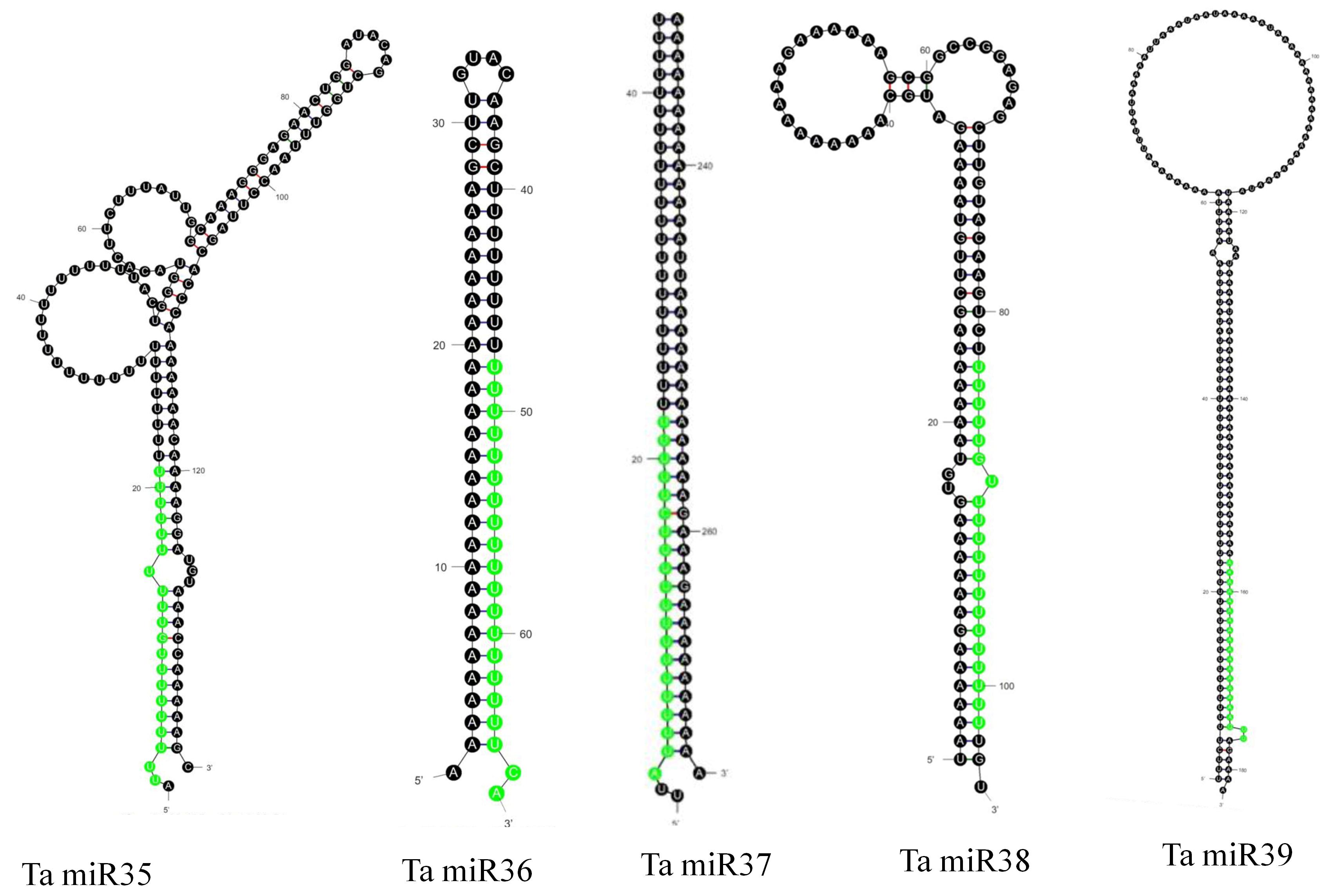


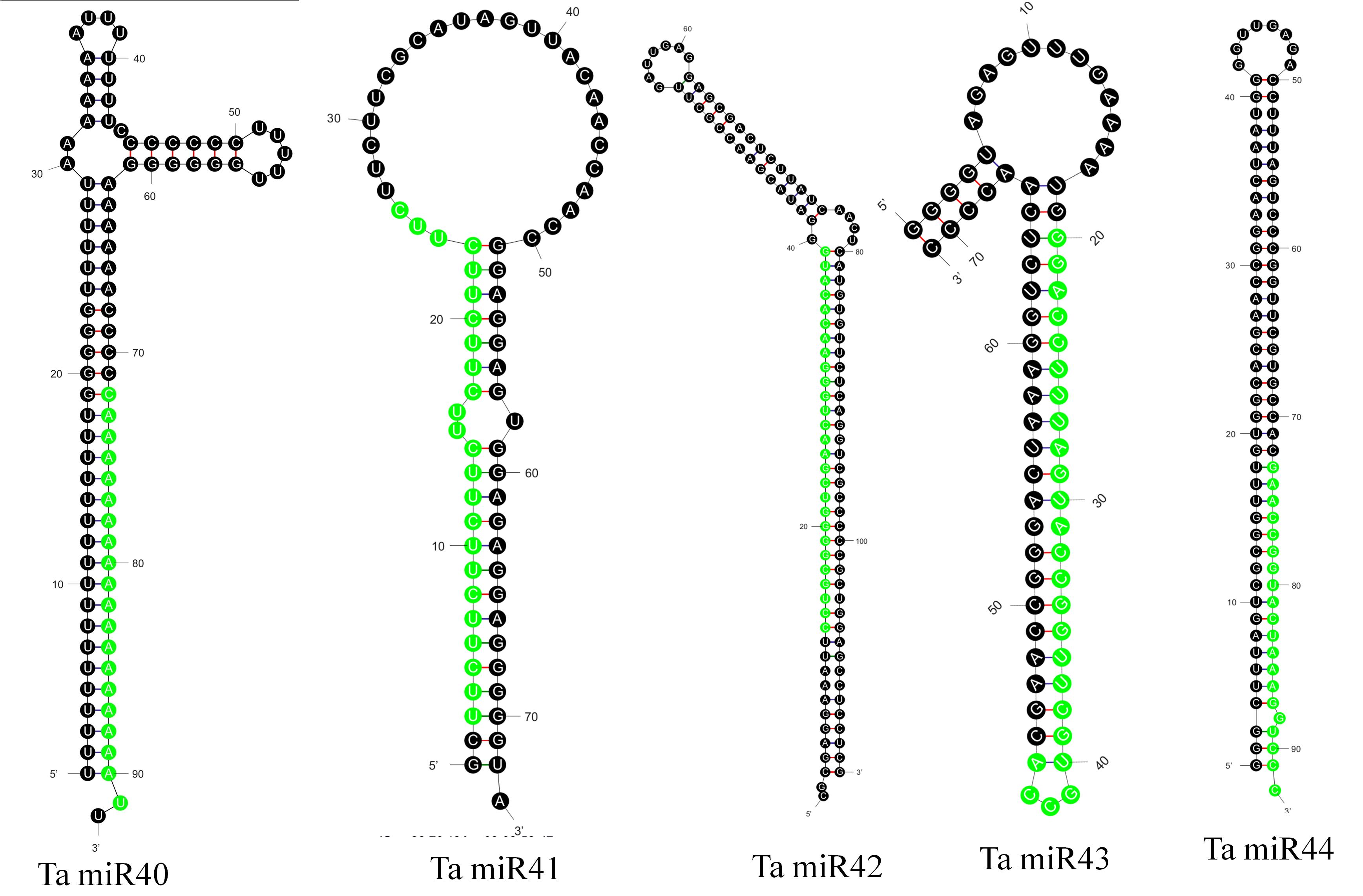


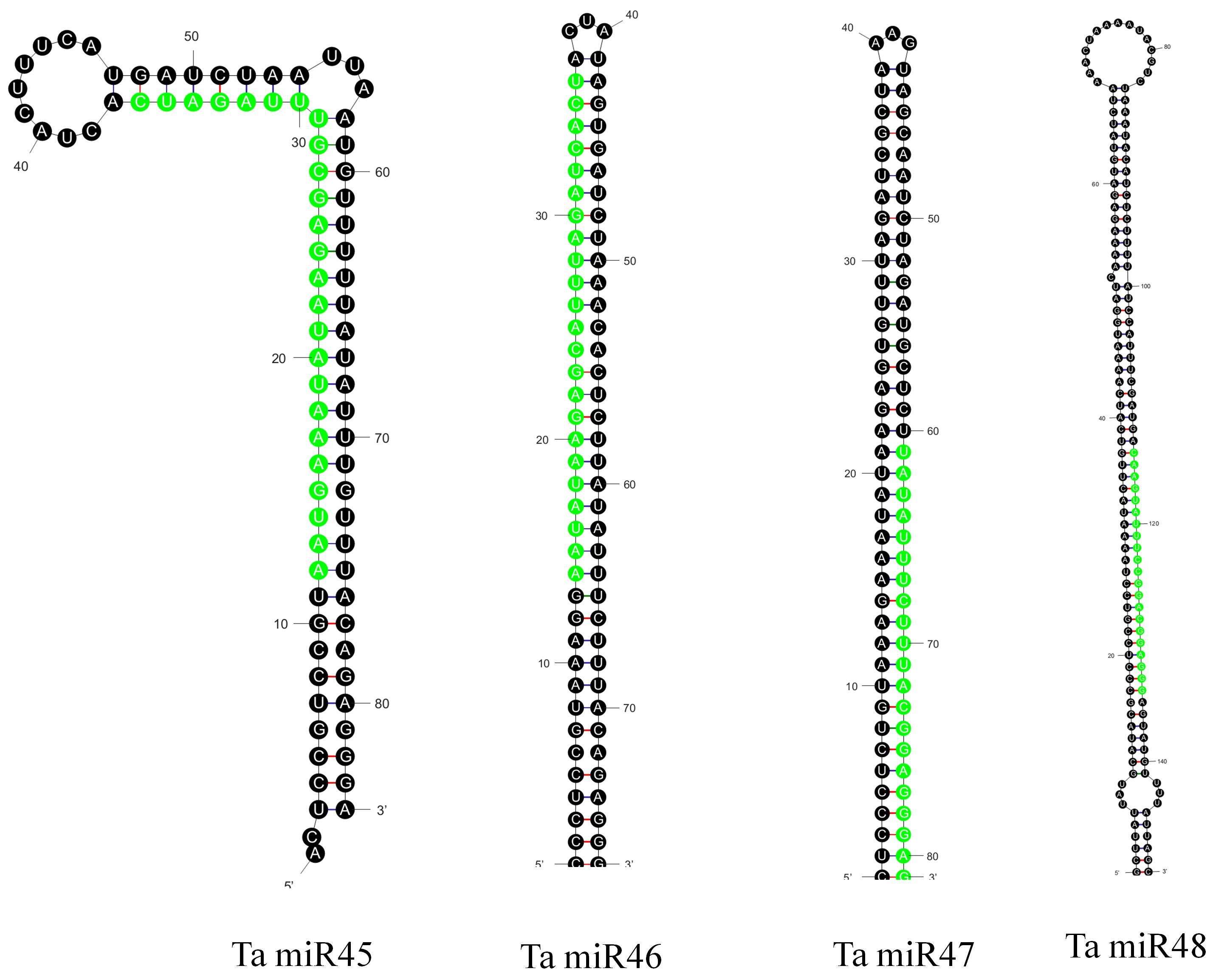


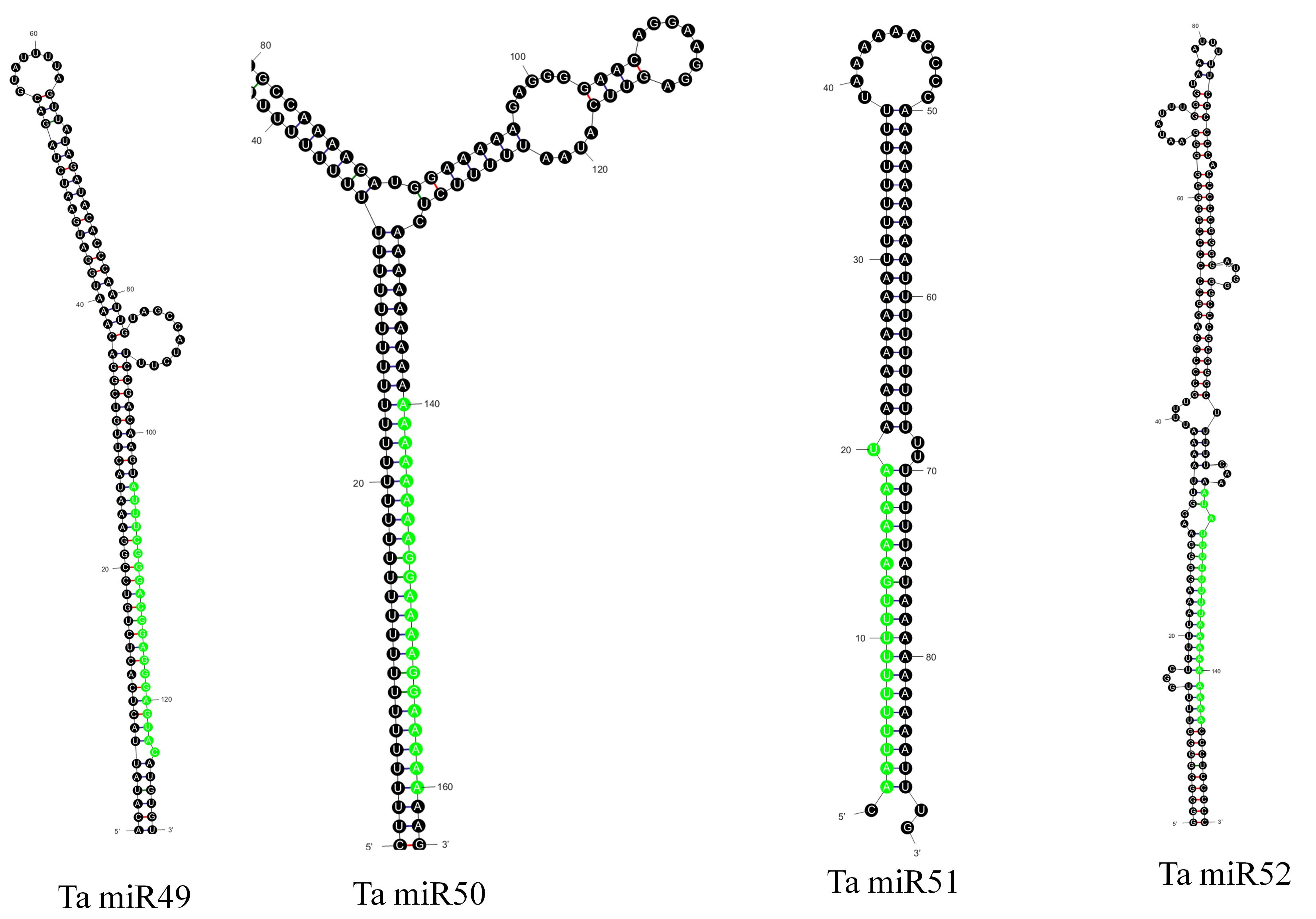


**Supplementary Fig. S1.** *Triticum aestivum* L*.* pre-miRNA secondary structures predicted using Mfold showing the mature miRNAs in stem portion (green).

**
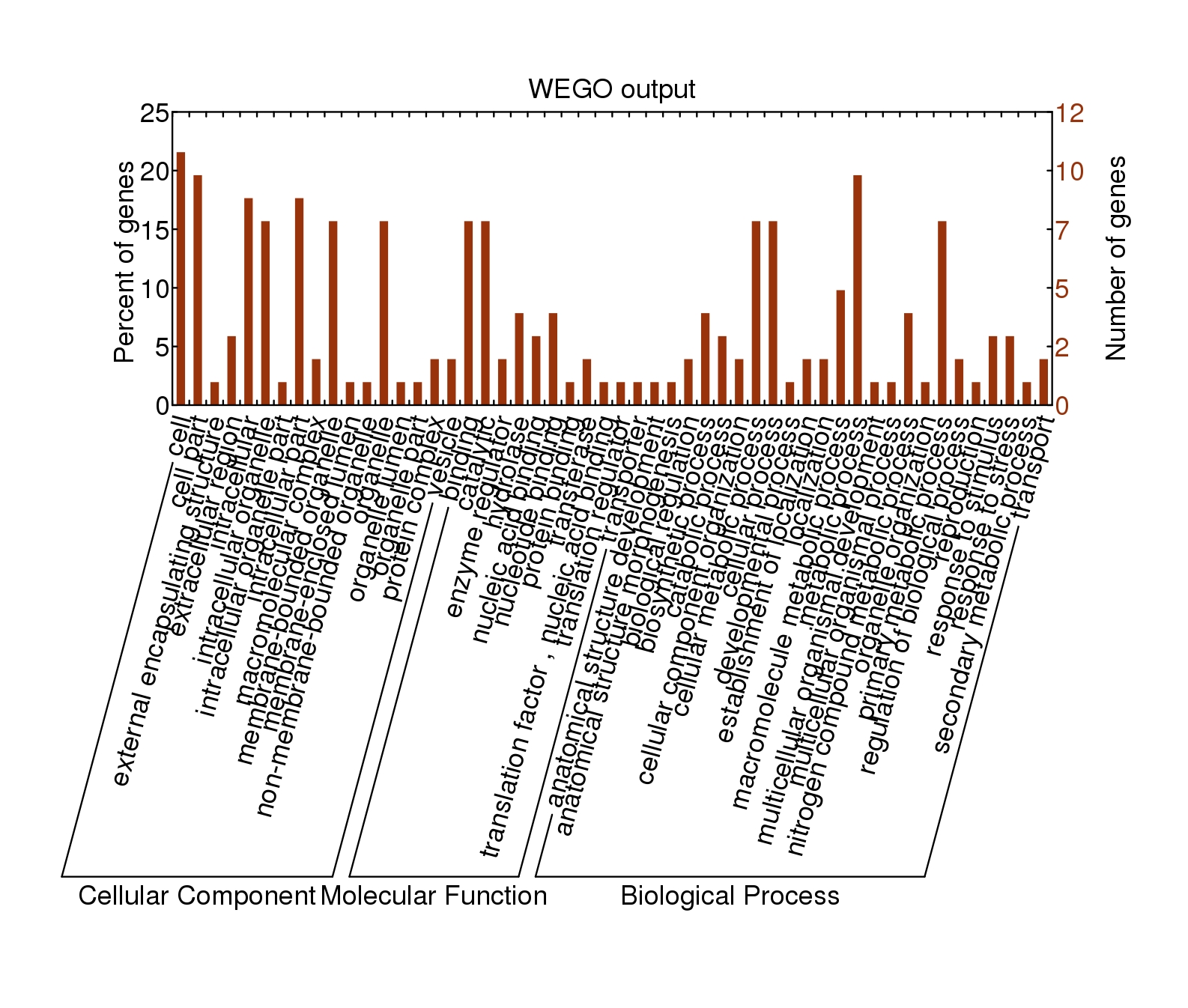
**

**Supplementary Fig. S2.** Gene Ontology (GO) categories and distribution of miRNA target genes in wheat. The results are classified in three main categories: cellular component, molecular function and biological process. The y-axis on the left indicates the percent of genes in a category, while the y-axis on the right indicates the number of genes in a specific category.


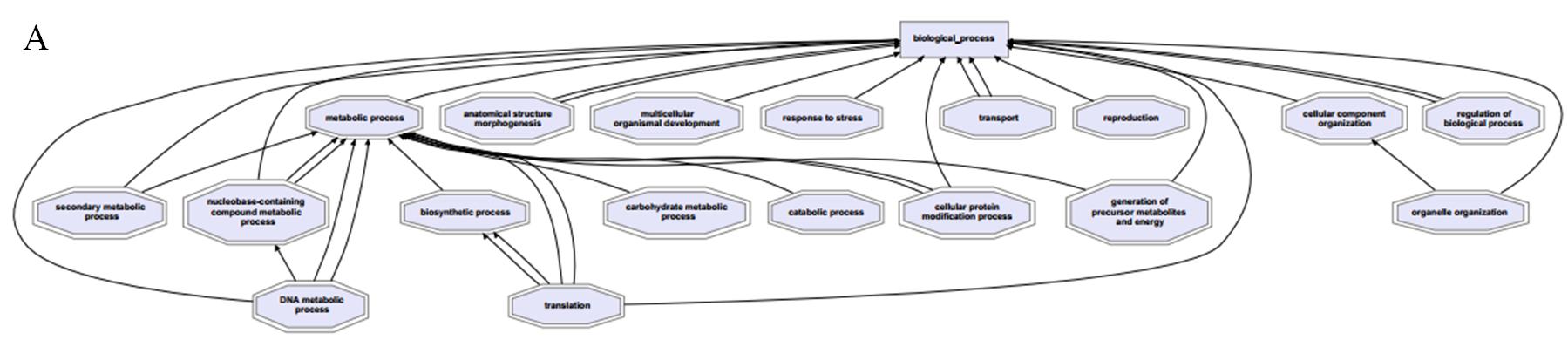


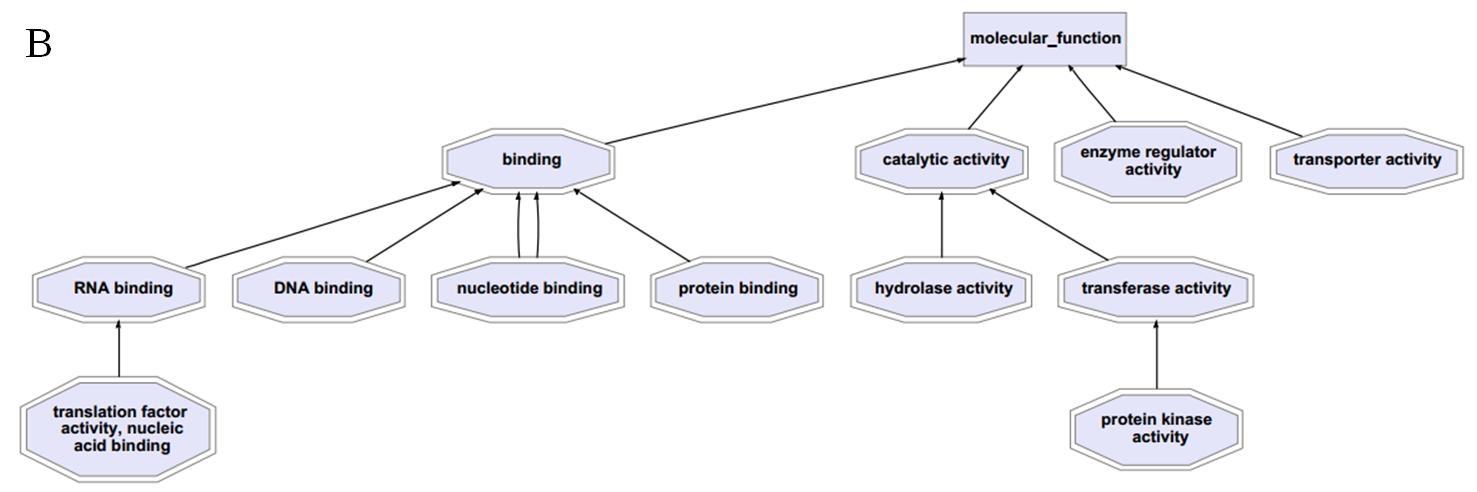


**Supplementary Fig. S3.** Gene Ontology (GO) term enrichment analysis of the miRNAs target genes. Analysis of the targetome of miRNAs within Biological process (A) and Molecular function (B) category. The predicted regulatory relationships between miRNA targets were performed by using the online tool Blast2GO with default parameters.

**Supplementary Table S1.** Wheat miRNAs identified by homolog search and secondary structure

| **Putative miRNA** | **Mature miRNA sequence** | **Length (nt)** | **Family** | **Tag counts** | | | | **ESTs ID** | **pre miRNA length (nt)** | **%** | | | | | | | | **∆G** | **AMFE** | **MFEI** |
| --- | --- | --- | --- | --- | --- | --- | --- | --- | --- | --- | --- | --- | --- | --- | --- | --- | --- | --- | --- | --- |
|  |  |  |  | **S-M** | **S-PI** | **R-M** | **R-PI** |  |  | **A** | **C** | **G** | **U** | **(A+U)** | **(G+C)** | **A/U** | **C/G** | **(MFE)** |  |  |
| TamiR 01 | UAUAUUAUGGGACGGAGGGAGUA | 23 | MIR818d | 5 | 0 | 0 | 0 | HX177233 | 72 | 33.33 | 19.44 | 19.44 | 27.77 | 61.1 | 33.33 | 1.2 | 1 | -31.2 | 43.33 | 1.11 |
| TamiR 02 | AAAUACUCCCUCCGUCCAAA | 20 | MIR1122 | 5 | 1 | 0 | 1 | CJ623282 | 112 | 35.71 | 19.64 | 17.85 | 26.68 | 62.39 | 37.5 | 1.33 | 1.1 | -30 | 26.78 | 0.71 |
| TamiR 03 | AAGUACUCCCUCCGUCCCAA | 20 |  | 0 | 1 | 0 | 0 | HX131010 | 91 | 32.96 | 20.87 | 14.28 | 31.86 | 64.82 | 35.2 | 1.03 | 1.46 | -43.2 | 47.47 | 1.34 |
| TamiR 04 | UUUUGGGACGGAGGGAGUACU # | 22 |  | 5 | 1 | 1 | 0 | HX189386 | 131 | 32.06 | 18.32 | 18.32 | 31.29 | 63.35 | 36.6 | 1.02 | 1 | -82.1 | 62.67 | 1.71 |
| TamiR 05 | UUACUCCCUCCGUCCCAAAAA | 21 |  | 0 | 2 | 1 | 0 | HX013753 | 129 | 33.33 | 17.05 | 16.27 | 33.33 | 66.66 | 33.32 | 1 | 0.87 | -50.1 | 38.83 | 1.16 |
| TamiR 06 | UCUAAAUACGGAUGUAUCAA | 20 |  | 6 | 0 | 0 | 0 | HX189386 | 142 | 31.69 | 16.9 | 17.6 | 33.8 | 65.49 | 29.1 | 0.94 | 1 | -81.1 | 38.54 | 1.32 |
| TamiR 07 | CUCCCUCUGUCCCAAAAUUCUUG # | 23 |  | 0 | 3 | 0 | 0 | HX045217 | 129 | 31 | 20.15 | 18.6 | 28.86 | 49.6 | 38.75 | 1..07 | 1.08 | -70.8 | 54.88 | 1.41 |
| TamiR 08 | UUGGGACGGAGGGAGUAUAAUU | 22 |  | 5 | 0 | 0 | 0 | CJ935515 | 115 | 26.08 | 19.13 | 15.65 | 37.39 | 63.47 | 34.8 | 0.69 | 1.22 | -23.1 | 20.08 | 0.57 |
| TamiR 09 | UUUUCGGGACGGAGGGAGUAU # | 22 |  | 4 | 5 | 3 | 1 | HX250719 | 99 | 32.32 | 17.17 | 18.18 | 32.32 | 64.64 | 35.35 | 1 | 0.94 | -30.6 | 30.9 | 0.87 |
| TamiR 10 | AGUAUUUUGGGACGGAGGGAG | 21 |  | 5 | 31 | 19 | 31 | HX078335 | 117 | 28.2 | 16.23 | 22.22 | 34.18 | 50.42 | 38.45 | 0.82 | 0.73 | -62.1 | 53.07 | 1.38 |
| TamiR 11 | AUAUUUAGGAACAGAGGGAGUA | 22 |  | 190 | 148 | 43 | 77 | HX006217 | 222 | 38.73 | 15.76 | 19.36 | 26.12 | 64.85 | 35.12 | 1.48 | 0.81 | -79.6 | 35.85 | 1.02 |
| TamiR 12 | UUAAUUCCGAACGGAGGGAG | 20 | MIR1127 | 0 | 0 | 0 | 5 | CJ849553 | 127 | 28.34 | 19.68 | 20.47 | 31.49 | 48.81 | 36.4 | 0.94 | 0.94 | -63.8 | 46.91 | 1.28 |
| TamiR 13 | UAUACUCCCUCCGUCCGGAA | 20 | MIR1133 | 0 | 5 | 0 | 5 | HX084785 | 105 | 25.71 | 19.04 | 19.04 | 36.19 | 61.9 | 38.08 | 0.71 | 1 | -38.9 | 37.04 | 0.97 |
| TamiR 14 | UCAUUUUGGGACGGAGGGAG | 20 | MIR1862 | 1 | 5 | 1 | 0 | FK827367 | 79 | 31.64 | 21.51 | 16.45 | 30.37 | 62.01 | 38 | 1.04 | 1.3 | -37.2 | 47.08 | 1.24 |
| TamiR 15 | UAUUUCGGACGGAGGGAGUA | 20 | MIR5490 | 0 | 0 | 5 | 0 | CD916693 | 106 | 27.35 | 17.92 | 22.64 | 32.07 | 59.42 | 40.6 | 0.85 | 0.79 | -44.3 | 41.79 | 1.03 |
| TamiR 16 | UCCGCCGCCGCCGCCGCCGC | 20 | MIR164f | 0 | 2 | 0 | 0 | HX135954 | 103 | 12.62 | 38.83 | 35.92 | 12.62 | 25.24 | 74.75 | 1 | 1.08 | -49.4 | 47.96 | 0.64 |
| TamiR 17 | CCCGCCGCCGCCGCCGCCGG | 20 | MIR168a | 0 | 5 | 4 | 0 | HX167489 | 71 | 8.45 | 39.43 | 33.8 | 18.3 | 26.75 | 73.23 | 0.46 | 1.16 | -27.2 | 38.3 | 0.52 |
| TamiR 18 | GGGAUCUCUAAAAAGACUUA # | 20 |  | 5 | 0 | 6 | 0 | BE415071 | 124 | 32.25 | 15.32 | 19.35 | 32.25 | 64.5 | 35 | 1 | 0.79 | -40.9 | 31.53 | 0.91 |
| TamiR 19 | AATTTTTTTTTTTTTTTTTTTTT | 23 | MIR169a | 1608 | 16508 | 675 | 1868 | GW667708 | 113 | 31.85 | 6.19 | 12.38 | 49.55 | 81.4 | 18.57 | 0.64 | 0.5 | -38.5 | 34.07 | 1.83 |
| TamiR 20 | TTTTTTTTTTTTTTTTTTTAAA | 22 |  | 54 | 224 | 25 | 68 | GW667707 | 119 | 23.52 | 25.21 | 21.84 | 29.41 | 52.93 | 47.05 | 0.79 | 1.15 | -51.2 | 42.81 | 0.91 |
| TamiR 21 | CTTTTTTTTTTTTTTTTTTTTT | 22 |  | 3116 | 25158 | 1179 | 3121 | JK972224 | 241 | 35.68 | 21.16 | 15.35 | 27.8 | 63.48 | 36.51 | 1.28 | 1.37 | -28.5 | 14.96 | 0.41 |
| TamiR 22 | ATTTTTTTTTTTTTTTTTTTTGAG | 24 |  | 144 | 680 | 56 | 80 | GD189163 | 140 | 25.57 | 12.85 | 27.85 | 33.35 | 58.92 | 40.7 | 0.76 | 0.46 | -38.9 | 27.78 | 0.68 |
| TamiR 23 | GTTTTTTTTTTTTTTTTTTTT | 21 |  | 29 | 155 | 9 | 11 | GW667658 | 59 | 38.98 | 10.17 | 13.56 | 37.29 | 76.27 | 23.73 | 1.05 | 0.75 | -28.3 | 49.35 | 2.08 |
| TamiR 24 | ATTTTTTTTTTTTTTTTTTTG | 21 |  | 4 | 11 | 1 | 4 | GH726135 | 660 | 33.18 | 17.87 | 22.72 | 26.21 | 59.39 | 40.6 | 1.26 | 0.78 | -143.4 | 21.52 | 0.53 |
| TamiR 25 | TTTTTTTTTTTTTTTTTTATG | 21 |  | 11 | 53 | 8 | 35 | GD186495 | 88 | 31.81 | 11.36 | 18.18 | 37.5 | 69.31 | 29.54 | 0.85 | 0.62 | -21.8 | 24.52 | 0.83 |
| TamiR 26 | TTTTTTTTTTTTTTTTTTGTG | 21 |  | 76 | 147 | 16 | 42 | GR303496 | 199 | 27.63 | 19.09 | 14.07 | 39.19 | 66.82 | 33.16 | 0.71 | 1.36 | -35.6 | 17.57 | 0.53 |
| TamiR 27 | TTTTTTTTTTTTTTTTTTTTG | 21 |  | 32 | 36 | 5 | 16 | GW667735 | 120 | 25 | 30.83 | 23.33 | 20 | 45 | 54.16 | 1.25 | 1.32 | -55.6 | 46.03 | 0.85 |
| TamiR 28 | TTTTTTTTTTTTTTTTTTGC | 20 |  | 1 | 7 | 1 | 2 | GD189776 | 111 | 29.72 | 13.51 | 19.81 | 36.93 | 66.65 | 33.33 | 0.8 | 0.68 | -20.6 | 18.33 | 0.55 |
| TamiR 29 | CATTTTTTTTTTTTTTTTTT | 20 |  | 81 | 439 | 30 | 44 | CO346143 | 227 | 33.48 | 16.29 | 20.7 | 29.51 | 62.99 | 37 | 1.13 | 0.78 | -44.9 | 19.61 | 0.53 |
| TamiR 30 | CGTTTTTTTTTTTTTTTTTT | 20 |  | 3 | 21 | 2 | 1 | DR737570 | 148 | 29.05 | 12.83 | 18.91 | 39.18 | 68.23 | 31.74 | 0.74 | 0.67 | -64.4 | 43.51 | 1.37 |
| TamiR 31 | TATTTTTTTTTTTTTTTTTG | 20 |  | 5 | 27 | 2 | 6 | DR732199 | 311 | 42.76 | 14.46 | 9 | 33.76 | 76.52 | 23.47 | 1.26 | 1.61 | -52.5 | 16.66 | 0.71 |
| TamiR 32 | TTTTTTTTTTTTTTTTGTGA | 20 |  | 93 | 487 | 30 | 68 | GR304307 | 234 | 27.35 | 21.36 | 14.52 | 36.75 | 64.1 | 35.89 | 0.74 | 1.47 | -32.21 | 13.63 | 0.38 |
| TamiR 33 | TTTTTTTTTTTTTTTTTAGA | 20 |  | 4 | 11 | 1 | 5 | GD187209 | 104 | 36.53 | 15.38 | 16.34 | 31.73 | 68.26 | 31.73 | 1.15 | 0.94 | -21.6 | 20.62 | 0.65 |
| TamiR 34 | TTTTTTTTTTTTTTGTTTTG | 20 |  | 4 | 40 | 7 | 18 | DR732340 | 99 | 28.28 | 20.2 | 12.12 | 39.39 | 67.67 | 32.32 | 0.72 | 1.67 | -17.4 | 17.45 | 0.54 |
| TamiR 35 | TTTTTTTTTGTTTTTTTTTT | 20 |  | 2 | 6 | 0 | 0 | GH727721 | 140 | 10.47 | 30.93 | 44.65 | 13.95 | 24.42 | 75.58 | 0.75 | 0.69 | -24.6 | 48.37 | 0.64 |
| TamiR 36 | TTTTTTTTTTTTTTTTTTCA | 20 |  | 1 | 16 | 0 | 0 | GD188827 | 67 | 46.27 | 5.97 | 4.48 | 43.28 | 89.56 | 10.45 | 1.06 | 1.33 | -26.6 | 39.71 | 3.8 |
| TamiR 37 | ATTTTTTTTTTTTTCTTTTT | 20 |  | 1 | 6 | 0 | 0 | DR732342 | 280 | 30.71 | 17.14 | 18.21 | 33.92 | 64.63 | 35.35 | 0.91 | 1.06 | -42.99 | 15.35 | 0.43 |
| TamiR 38 | TTTTTGTTTTTTTTTTTTTT | 20 |  | 3 | 16 | 0 | 0 | GD189818 | 105 | 42.86 | 7.62 | 18.1 | 31.43 | 74.29 | 25.71 | 1.36 | 0.42 | -23.2 | 21.85 | 0.85 |
| TamiR 39 | AACTTTTTTTTTTTTTTTTT | 20 |  | 2 | 6 | 0 | 0 | DR732330 | 241 | 35.68 | 21.16 | 15.35 | 27.8 | 63.48 | 36.51 | 1.28 | 1.37 | -39.03 | 16.19 | 0.44 |
| TamiR 40 | ATTTTTTTTTTTTTTTTTTG | 20 | MIR396a | 3 | 33 | 0 | 0 | DR737503 | 92 | 33.7 | 13.04 | 11.96 | 41.3 | 75 | 25 | 0.82 | 1.09 | -43.2 | 46.75 | 1.87 |
| TamiR 41 | UUCUUCUUCUUCUUCUUCUUCUUC | 24 | MIR166a | 4 | 6 | 0 | 0 | HX167182 | 72 | 16.66 | 23.61 | 25 | 34.72 | 51.38 | 48.6 | 0.47 | 0.94 | -23.7 | 32.92 | 0.67 |
| TamiR 42 | UGCGGGGUCGAGCUGGGAACACAU | 24 | MIR398 | 4 | 5 | 5 | 0 | CJ711035 | 116 | 20.68 | 28.84 | 29.31 | 21.55 | 42.41 | 58.15 | 0.95 | 0.98 | -57.1 | 49.22 | 0.84 |
| TamiR 43 | GGACCUUUAGUACCGGUUCGUGCCA | 25 | MIR1117 | 6 | 3 | 0 | 0 | CA697217 | 71 | 25.35 | 23.94 | 29.57 | 21.12 | 46.47 | 53.51 | 1.2 | 0.8 | -38.2 | 53.8 | 1 |
| TamiR 44 | GAACCGGUACUAAAGGUCCCAUU # | 23 |  | 1 | 0 | 0 | 0 | HX119495 | 99 | 19.19 | 25.25 | 29.29 | 26.26 | 45.45 | 54.5 | 0.73 | 0.86 | -53 | 54.94 | 1 |
| TamiR 45 | GAUCUAAACGCUCUUAUAUUUCAUU | 25 | MIR1121 | 5 | 5 | 0 | 0 | CA653878 | 84 | 30.95 | 14.28 | 16.66 | 38.09 | 69.04 | 21.2 | 0.73 | 0.75 | -24.3 | 26.06 | 1.22 |
| TamiR 46 | AGUGAUCUAAAUGCUCUUAUAUU | 23 |  | 5 | 5 | 2 | 2 | HX165205 | 81 | 33.33 | 19.75 | 16.04 | 30.86 | 64.19 | 36.4 | 1.08 | 1.23 | -40 | 54.32 | 1.49 |
| TamiR 47 | AUAUUUCUUUACAGAGGGAGUAC | 23 |  | 4 | 2 | 5 | 3 | HX138159 | 88 | 32.95 | 14.77 | 20.45 | 31.81 | 64.76 | 35.2 | 1.03 | 0.72 | -50.5 | 59.2 | 1.68 |
| TamiR 48 | CAAGUAUUUCCGGACGGAGGG | 21 | MIR1128 | 0 | 0 | 0 | 5 | HX129089 | 150 | 32.66 | 19.33 | 16 | 32 | 64.66 | 35.33 | 1.2 | 1.2 | -70 | 46.66 | 1.32 |
| TamiR 49 | CGUACUCCCUCCGUCCCGAAAU | 22 | MIR1862a | 16 | 5 | 2 | 2 | CA611717 | 130 | 29.23 | 19.23 | 21.53 | 30 | 59.23 | 40.76 | 0.97 | 0.89 | -51.9 | 39.92 | 0.98 |
| TamiR 50 | UUUUUCCUUUUCCUUUUUUUU | 21 | MIR5149 | 7 | 0 | 0 | 0 | DR735117 | 163 | 39.87 | 5.52 | 13.49 | 41.1 | 80.97 | 19 | 0.97 | 0.41 | -36.5 | 22.39 | 1.18 |
| TamiR 51 | AAUUUUUUUUUGAAAAAAUU | 20 | MIR812j | 0 | 6 | 0 | 0 | CK198340 | 89 | 47.19 | 5.6 | 2.22 | 44.94 | 92.13 | 7.9 | 1.05 | 2.5 | -22.1 | 24.83 | 3.14 |
| TamiR 52 | UUUUUUUUUAAAAAAAAUAU | 20 | MIR1877 | 2 | 13 | 4 | 2 | DR735657 | 153 | 20.91 | 22.87 | 29.41 | 26.79 | 47.7 | 52.3 | 0.78 | 0.77 | -72.8 | 48.69 | 0.93 |

# miRNAs in antisense orientation; First Fourteen miRNAs are conserved; TamiR15 and TamiR16 reported for the first time; MFE: Minimal folding free energy; MFEI: Minimal folding free energy index; AMFE: Adjusted minimal folding free energy

**Supplementary Table S2.** Potential target genes for the identified miRNAs

| **Putative miRNA** | **Target_Acc.** | **Target Description** |
| --- | --- | --- |
| TamiR01 | CJ542241 | Co-expression of het-e and het-c lead to cell death |
|  | TC417827 | Os05g0304100 protein |
|  | TC429127 | Phosphate-regulating neutral endopeptidase (Metalloendopeptidase homolog PEX) (X-linked hypophosphatemia protein) (HYP) (Vitamin D-resistant hypophosphatemic rickets protein) |
| TamiR02 | TC368568 | SET domain protein |
|  | CJ612181 | Os03g0156800 protein |
|  | TC386110 | Glycosylphosphatidylinositol anchor attachment 1 protein |
|  | CK216914 | AGAP000043-PA |
| TamiR03 | CN013262 | CG13742-PA |
|  | TC402807 | Os08g0344700 protein |
|  | TC381627 | Cinnamyl-alcohol dehydrogenase-like protein |
|  | TC412501 | Transcriptional regulator, TetR family |
| TamiR04 | TC393171 | Homologue to emb|Z00028.1|CHZMRRNA Zea mays chloroplast rRNA-operon |
|  | TC412501 | Transcriptional regulator, TetR family |
|  | TC450163 | Subtilisin-like protein |
|  | BJ323295 | Receptor protein kinase |
| TamiR05 | CJ868604 | Transcriptional activator-like |
|  | TC439689 | *Calreticulin-like protein* |
|  | TC461453 | Irvingia malayana 18S ribosomal RNA gene |
|  | BJ323295 | Receptor protein kinase |
| TamiR06 | TC371963 | Alpha-1,2-fucosidase |
|  | CJ868604 | Transcriptional activator-like |
|  | TC405483 | Similar to chr5 scaffold_2, whole genome shotgun sequence Vitis vinifera |
|  | TC397544 | Acetyl-CoA C-acetyltransferase |
|  | TC422344 | Single-stranded DNA-binding protein, mitochondrial precursor |
|  | TC424098 | Metallo-beta-lactamase-like |
|  | BQ753167 | PREDICTED: splicing factor 1 isoform 1 |
|  | CK194884 | Sucrase-like protein |
| TamiR07 | TC384315 | Os02g0225300 protein |
|  | DR731536 | DEAD-box ATP-dependent RNA helicase 50 |
|  | CJ685596 | UDP-N-acetylmuramoyl-L-alanyl-D-glutamate--2,6-diaminopimelate ligase |
|  | CJ554691 | Phosphoenolpyruvate synthase |
|  | TC444618 | Abscisic stress ripening-like protein |
|  | TC393171 | Homologue to emb\|Z00028.1\|CHZMRRNA Zea mays chloroplast rRNA-operon |
|  | TC412501 | Transcriptional regulator, TetR family |
|  | TC450163 | Subtilisin-like protein |
|  | BJ323295 | Receptor protein kinase |
| TamiR08 | TC383543 | Citrate synthase |
|  | TC409271 | CDK5 activator-binding protein-like |
|  | DR731536 | DEAD-box ATP-dependent RNA helicase 50 |
|  | TC405893 | Os03g0860000 protein |
|  | TC394286 | CTV.22 |
| TamiR09 | TC397848 | Expressed protein |
|  | TC412501 | Transcriptional regulator, TetR family |
|  | TC450163 | Subtilisin-like protein |
|  | TC411759 | Homologue to chr11 scaffold_14, whole genome shotgun sequence Vitis vinifera |
| TamiR10 | CD896057 | Ferredoxin-NADP reductase precursor |
|  | TC401785 | Urocortin precursor |
|  | TC457394 | Gll1679 protein |
|  | TC438389 | Peptide-acetyl-coenzyme A transporter (PAT) family protein |
|  | TC409271 | CDK5 activator-binding protein-like |
| TamiR11 | TC405029 | DNA polymerase III delta subunit |
|  | CD891662 | F-box protein-like |
|  | TC438636 | Mitochondrial phosphate transporter |
|  | CA611067 | Glutathione-S-transferase 28e45 |
| TamiR12 | TC422344 | Single-stranded DNA-binding protein, mitochondrial precursor |
|  | TC424098 | Metallo-beta-lactamase-like |
|  | BQ753167 | PREDICTED: splicing factor 1 isoform 1 |
|  | CK194884 | Sucrase-like protein |
| TamiR13 | TC432967 | Predicted protein |
|  | TC394098 | Protein kinase domain containing protein, expressed |
|  | BE516586 | Calmodulin-like protein |
|  | DR736559 | Zinc finger protein 628 |
| TamiR14 | TC376766 | DEAD-box ATP-dependent RNA helicase 42 |
|  | TC459951 | Protein kinase domain containing protein, expressed |
|  | TC446878 | Os04g0553300 protein |
|  | TC383543 | Citrate synthase |
| TamiR15 | CJ562562 | Phytase |
|  | TC390088 | Coatomer subunit zeta-1 |
|  | CJ868699 | NADH-ubiquinone/plastoquinone oxidoreductase, chain 6 precursor |
|  | TC374770 | DEAD-box ATP-dependent RNA helicase 31 |
|  | CK207823 | Kelch motif family protein, expressed |
| TamiR16 | TC371370 | Os11g0104200 protein |
|  | TC436071 | 2-alkenal reductase |
|  | CV780255 | Leucine-rich repeat family protein-like |
|  | TC455984 | Glutamine amidotransferase, class I, active site |
| TamiR17 | EF368361 | WRKY10 |
|  | AY781349 | DREB3 |
|  | DQ512340 | MADS Box |
|  | DQ512335 | TaAGL18 |
| TamiR18 | CA683464 | Major facilitator superfamily MFS_1 |
|  | GH727822 | Mpp10 protein, expressed |
|  | TC388450 | Delta-aminolevulinic acid dehydratase, chloroplast precursor |
|  | TC424098 | Metallo-beta-lactamase-like |
|  | CA601255 | Membrane bound O-acyl transferase MBOAT |
| TamiR19 | TC368638 | DREB transcription factor 4A |
|  | TC368658 | DREB transcription factor 4B |
|  | TC401738 | Sec1 precursor |
|  | TC385570 | Universal stress protein / early nodulin ENOD18-like |
|  | TC389125 | Plastidic phosphate translocator-like protein2 |
|  | TC445341 | Low molecular weight glutenin |
|  | TC434974 | Cysteine synthase (O-acetylserine sulfhydrylase) (O- acetylserine (Thiol)-lyase |
|  | TC453828 | S-adenosylmethionine decarboxylase |
|  | TC456515 | Dehydrin 13 |
|  | TC461940 | Adenosine kinase-like protein |
|  | CV761287 | Glycosyltransferase |
|  | TC384928 | Ribosomal Pr 117 |
|  | TC455998 | Chromosome segregation ATPases-like protein |
|  | CK166267 | M.musculus 45S pre rRNA gene |
| TamiR20 | TC404101 | Endo-1,4-beta-glucanase |
|  | TC447609 | Protein kinase domain containing protein, expressed |
|  | TC369359 | Vacuolar ATP synthase subunit E |
|  | NP9351886 | cytochrome P450-like protein |
|  | TC405735 | PHD zinc finger protein |
|  | TC430458 | Zinc finger transcription factor ZFP30 |
|  | TC386248 | BolA-like |
|  | TC438964 | Adenosylhomocysteinase |
|  | TC373975 | MBD5 |
|  | TC441169 | Adenosylhomocysteinase |
|  | TC449119 | Glutathione transferase F4 |
|  | DR740043 | Asense-like protein |
|  | TC456212 | Phospholipase D |
|  | DR741931 | Serine/threonine kinase; n=1 |
| TamiR21 | TC371388 | Starch branching enzyme IIb |
|  | TC447609 | Protein kinase domain containing protein, expressed |
|  | TC428540 | Inducible phenylalanine ammonia-lyase |
|  | TC436861 | NAD(P)H-quinone oxidoreductase chain 4, chloroplast (NAD(P)H dehydrogenase, chain 4), NAD(P)H-quinone oxidoreductase chain 4, chloroplast (NAD(P)H dehydrogenase, chain 4 |
|  | TC370678 | Calcium-dependent protein kinase 2 |
|  | TC412906 | GDSL-motif lipase/hydrolase-like |
|  | TC391347 | s-locus protein 5 |
|  | TC373973 | Type 1 non-specific lipid transfer protein precursor |
|  | TC374016 | Predicted protein |
|  | TC459454 | Quinone reductase |
|  | TC459454 | Quinone reductase |
|  | TC455152 | Ribulose bisphosphate carboxylase small chain |
|  | TC454756 | Cold-responsive protein COR14a |
|  | TC383617 | Peroxidase 8 |
| TamiR22 | TC400103 | Photosystem I reaction center subunit XI, chloroplast precursor |
|  | TC455271 | rRNA intron-encoded homing endonuclease |
|  | TC458815 | Ribulose bisphosphate carboxylase small chain clone 512 |
|  | TC429121 | Gamma-thionin |
|  | BQ609635 | Ribosomal protein L35A |
|  | TC425867 | Ribosomal protein |
|  | TC427775 | UMP synthase |
|  | DR731677 | Predicted protein |
|  | TC437535 | Histone H3 |
|  | TC436448 | PDI-like protein |
|  | TC390415 | Chlorophyll a/b-binding protein WCAB precursor |
|  | TC460477 | Predicted protein |
| TamiR23 | TC394189 | Expressed protein |
|  | TC369053 | DREB transcription factor 6 |
|  | TC369359 | Vacuolar ATP synthase subunit E |
|  | TC460779 | LacZ alpha peptide |
|  | TC373621 | GTP-binding protein TypA |
|  | TC368660 | Floral homeotic protein |
|  | TC455242 | Histone H2A.2.2 |
|  | TC400103 | Photosystem I reaction center subunit XI, chloroplast precursor |
|  | TC437449 | 60S ribosomal protein L10-2 |
|  | TC457259 | Ribulose bisphosphate carboxylase small chain |
|  | TC460772 | Probable histone H2A.5 |
|  | TC443246 | Structural polyprotein |
|  | TC423283 | Photosystem II core complex proteins psbY, chloroplast precursor (L- arginine-metabolizing enzyme) (L-AME) [Contains: Photosystem II protein psbY-1 (psbY-A1); Photosystem II protein psbY-2 (psbY-A2)] |
|  | TC460357 | Clathrin coat assembly protein |
|  | TC460477 | Predicted protein |
|  | TC457094 | Bp5A protein |
|  | TC397977 | Glycoprotein |
|  | TC446383 | Precursor of CP29, core chlorophyll a/b binding (CAB) protein of photosystem II |
|  | TC413086 | GCK-like kinase MIK |
|  | TC455452 | DEAD-box ATP-dependent RNA helicase 5 |
|  | TC455271 | rRNA intron-encoded homing endonuclease |
| TamiR24 | DR733611 | Aleurone ribonuclease |
|  | BQ244229 | Chromosome segregation ATPases-like protein |
|  | DR736152 | Plasma membrane intrinsic protein |
|  | TC426091 | Histone H3.2 |
|  | CA736350 | Chromosome segregation ATPases-like protein |
|  | BQ236337 | Chromosome segregation ATPases-like protein |
|  | TC371388 | Starch branching enzyme IIb; n=3; Triticeae |
|  | TC379134 | Polypyrimidine tract-binding protein 1-like |
| TamiR25 | TC436122 | Flavonoid O-methyltransferase |
|  | BE418178 | SNF7 family protein, expressed |
|  | CK217228 | 40S ribosomal protein S29 |
|  | CV773859 | Seed maturation protein |
|  | CV064626 | Chromosome segregation ATPases-like protein |
|  | TC460890 | Cysteine protease |
|  | TC368694 | RAFTIN1a protein |
|  | TC427978 | Protein H2A.6 |
|  | TC432673 | Pherophorin-dz1 protein precursor |
|  | CK206604 | Membrane protein |
|  | TC456515 | Dehydrin 13 |
| TamiR26 | TC401738 | Sec1 precursor |
|  | TC372573 | Serine/threonine protein phosphatase |
|  | CK210535 | Beta-D-xylosidase |
|  | CK214236 | Ribulose bisphosphate carboxylase small chain |
|  | CK214235 | Ribulose bisphosphate carboxylase small chain |
|  | CV772763 | Glycosyltransferase |
|  | TC399912 | Expressed protein |
|  | CK157964 | IQ motif containing D |
|  | TC452537 | Glycosyltransferase |
|  | TC437017 | Alpha-gliadin Gli2-LM2-12 |
|  | NP799404 | G protein alpha subunit |
|  | TC457259 | Ribulose bisphosphate carboxylase small chain |
| TamiR27 | TC371388 | Starch branching enzyme IIb |
|  | TC379134 | Polypyrimidine tract-binding protein 1-like |
|  | TC368638 | DREB transcription factor 4A |
|  | TC368646 | DREB transcription factor 4A |
|  | TC368658 | DREB transcription factor 4B |
|  | TC454324 | 26S proteasome regulatory particle non-ATPase subunit12 |
|  | TC431647 | Chlorophyll a/b-binding protein CP29 precursor |
|  | TC368660 | Floral homeotic protein |
|  | TC436448 | PDI-like protein |
|  | NP799404 | G protein alpha subunit |
|  | TC417747 | Adenyl cyclase |
|  | TC455152 | Ribulose bisphosphate carboxylase small chain |
| TamiR28 | TC379134 | Polypyrimidine tract-binding protein 1-like |
|  | TC368660 | Floral homeotic protein |
|  | TC457259 | Ribulose bisphosphate carboxylase small chain |
|  | TC378798 | Alpha gliadin |
|  | TC439938 | Ribulose bisphosphate carboxylase small chain |
|  | TC371557 | NB-ARC domain containing protein, expressed |
|  | TC459712 | Cysteine proteinase inhibitor |
|  | CK210933 | Polyubiquitin |
|  | TC410459 | Beta3-glucuronyltransferase |
|  | TC379027 | Photosystem 1 subunit 5 |
|  | TC377481 | Cold induced protein-like |
|  | CK206334 | Histone H1 WH1A.4 |
| TamiR29 | BQ244229 | Chromosome segregation ATPases-like protein |
|  | CK196042 | Diadenosine 5',5'''-P1,P4-tetraphosphate hydrolase |
|  | DR741561 | Glycine-rich RNA-binding protein |
|  | TC440572 | Organic cation transporter-like protein |
|  | CK214284 | Ribulose bisphosphate carboxylase small chain |
| TamiR30 | CK210310 | Guanine nucleotide-binding protein subunit beta-like protein |
|  | TC398094 | Aspartyl-tRNA synthetase |
|  | CV766777 | Metallothionein-like protein 1 |
|  | TC400069 | rypsin/alpha-amylase inhibitor CMX1/CMX3 precursor |
|  | TC456294 | Avenin-like protein |
|  | TC450972 | Early nodulin |
|  | TC412961 | Chromosome segregation ATPases-like protein |
|  | CK159742 | Glycine-rich cell wall protein precursor |
|  | CK198448 | Tetratricopeptide TPR_4 precursor |
|  | TC457068 | Riboflavin synthase alpha chai |
|  | BE516232 | Type 1 non-specific lipid transfer protein precursor |
| TamiR31 | DR737575 | Peptide mating pheromone Bbp2-2 |
|  | BQ609634 | Ribosomal protein L18a-like |
|  | TC436448 | PDI-like protein |
|  | TC378798 | Alpha gliadin |
|  | TC444510 | Ubiquitin carboxyl-terminal hydrolase |
|  | TC432673 | Pherophorin-dz1 protein precursor |
|  | TC460607 | Chromosome segregation ATPases-like protein |
| TamiR32 | TC401738 | Sec1 precursor |
|  | TC420636 | Photosystem I reaction center subunit psaK, chloroplast precursor |
|  | CK217194 | WCOR719 |
|  | CK214236 | Ribulose bisphosphate carboxylase small chain |
|  | CK214235 | Ribulose bisphosphate carboxylase small chain |
|  | TC450082 | SJCHGC09076 protein |
|  | TC373468 | Low molecular mass early light-inducible protein HV90, chloroplast precursor |
|  | CK209959 | Temperature stress-induced lipocalin |
|  | TC436122 | Flavonoid O-methyltransferase |
|  | TC375056 | Glycine-rich RNA-binding protein |
| TamiR33 | TC369053 | DREB transcription factor 6 |
|  | TC455242 | Histone H2A.2.2 |
|  | NP410206 | isoamylase precursor; glycogen 6-glucanohydrolase |
|  | TC445341 | Low molecular weight glutenin |
|  | TC397977 | Glycoprotein |
|  | TC431432 | Amidase family protein, expressed |
|  | TC407558 | Thioredoxin H-type |
|  | TC372855 | Ubiquitin carrier protein |
|  | TC381532 | NADH-ubiquinone oxidoreductase 20 kDa subunit, mitochondrial precursor |
|  | TC434025 | Alpha tubulin-2A |
| TamiR34 | CK209325 | Chitinase 3 |
|  | GH727704 | Cell divisionFtsK/SpoIIIE |
|  | TC373168 | Atency associated nuclear antigen-like |
|  | DR737090 | Cell wall protein pherophorin-C10 |
|  | TC454324 | 26S proteasome regulatory particle non-ATPase subunit12 |
|  | TC455152 | Ribulose bisphosphate carboxylase small chain |
|  | TC389125 | Plastidic phosphate translocator-like protein2 |
|  | TC460607 | Chromosome segregation ATPases-like protein |
| TamiR35 | TC454324 | 26S proteasome regulatory particle non-ATPase subunit12 |
|  | DR735553 | Cleavage and polyadenylation specificity factor subunit 6 |
|  | DR736194 | Chromosome segregation ATPases-like protein |
|  | DR741089 | Peptidase S1 and S6, chymotrypsin/Hap precursor |
|  | DR740971 | Ras-related protein Rab11B |
|  | TC459987 | Ubiquitin fusion protein; n=3 |
|  | CV766324 | Chromosome segregation ATPases-like protein |
|  | CV772693 | PE-PGRS family protein |
| TamiR36 | BQ243047 | Hydroxyproline-rich protein |
|  | CA736863 | Sel1 precursor |
|  | TC451701 | beta-galactosidase |
|  | CV779129 | beta-galactosidase |
|  | TC450966 | Peroxidase 8 |
|  | CA595321 | Phosphorylase |
|  | CV781457 | BLT14.1 protein |
|  | BE516151 | Elongation factor |
|  | TC457104 | 6,7-dimethyl-8-ribityllumazine synthase |
|  | TC461210 | beta-galactosidase |
|  | TC455006 | Pseudouridine synthase |
| TamiR37 | CK209902 | IDS1 protein |
|  | DR734855 | Fus-like protein |
|  | CK195779 | Probable acetyltransferases |
|  | TC452289 | Chromosome segregation ATPases-like protein |
|  | TC446383 | Precursor of CP29, core chlorophyll a/b binding (CAB) protein of photosystem II |
|  | TC433384 | Proline-rich protein |
|  | DR737845 | Chromosome segregation ATPases-like protein |
|  | DR735621 | Chromosome segregation ATPases-like protein |
|  | CK162975 | Microtubule-binding protein TANGLED1 |
|  | TC455963 | Monodehydroascorbate reductase |
|  | TC459461 | Pistil extensin-like protein |
|  | CV775479 | Heavy metal transporter MTP1 |
| TamiR38 | DR735553 | Cleavage and polyadenylation specificity factor subunit 6 |
|  | TC459712 | Cysteine proteinase inhibitor |
|  | DR739057 | Histone H2B.1 |
|  | TC371544 | Superal1; n=1 |
|  | DR740971 | Ras-related protein Rab11B |
|  | TC459987 | Ubiquitin fusion protein; n=3 |
|  | CV772763 | Glycosyltransferase |
|  | CV780653 | Metallothionein-like protein 1 |
| TamiR39 | CK160047 | Collagen alpha-2(XI) chain precursor |
|  | BE418790 | Ferredoxin-NADP(H) oxidoreductase |
|  | CK201275 | Meltrins, fertilins and related Zn-dependent metalloproteinases of the ADAMs family |
|  | CK156516 | Chromosome segregation ATPases-like protein |
|  | BQ242645 | SnoRNP component, Nop56p/58p homolog |
|  | BG904628 | PE-PGRS family protein |
|  | TC390033 | Ethylene-responsive element binding protein 2 |
|  | TC427978 | Protein H2A.6 |
|  | TC436122 | Flavonoid O-methyltransferase |
|  | CV064399 | ABC transporter related |
|  | TC448277 | Polylysine protein precursor |
| TamiR40 | CV066241 | Transcription initiation factor TFIID subunit |
|  | CK166499 | Extensin |
|  | CK160026 | Beta-galactosidase |
| TamiR41 | AJ888618 | Zinc finger protein 428 |
|  | CD895848 | Predicted protein |
|  | CA714803 | Peptidase S1 and S6, chymotrypsin/Hap precursor |
|  | GH726983 | Membrane protein |
|  | CJ527937 | SJCHGC01957 protein |
| TamiR42 | CJ813940 | Similar to chr7 scaffold_42, whole genome shotgun sequence Vitis vinifera |
|  | CA681257 | Carotenoid cleavage dioxygenase 4a |
|  | TC385713 | Glutamine synthetase |
| TamiR43 | TC435101 | Myosin VIII ZMM3 |
| TamiR44 | TC390649 | Pr1 |
|  | TC381774 | Weakly similar to chromosome undetermined scaffold_133, whole genome shotgun sequence Vitis vinifera |
| TamiR45 | CA653878 | Beta-glucanase |
|  | TC414646 | Peroxidase 6 |
|  | CJ839181 | Ubiquitin carrier protein |
|  | TC375411 | Glycerol kinase-like protein |
|  | CA654281 | FecR protein |
| TamiR46 | BE516431 | TPR Domain containing protein |
|  | CA712284 | D-amino acid oxidase |
|  | CK194611 | NAD-dependent epimerase/dehydratase |
|  | CA653878 | Beta-glucanase |
|  | TC428723 | Similar to chr3 scaffold_8, whole genome shotgun sequence - Vitis vinifera |
| TamiR47 | TC414908 | Metallothionein-like protein 4A |
|  | TC444828 | Polyubiquitin |
|  | TC399039 | Fructose-1,6-bisphosphatase 2 |
|  | BE516431 | TPR Domain containing protein, expressed |
|  | TC389233 | 6-4 photolyase |
| TamiR48 | CJ560586 | Ribosome recycling factor, chloroplast precursor |
|  | CK192977 | Os03g0621400 protein |
|  | CK159384 | Ubiquitin carrier protein |
|  | TC383624 | Peroxidase 6 |
|  | TC405193 | Os05g0556100 protein |
|  | TC394098 | Protein kinase domain containing protein, expressed |
|  | BE516586 | Calmodulin-like protein |
|  | DR736559 | Zinc finger protein 628 |
| TamiR49 | TC447824 | Transcriptional activator of nitrogen-regulated genes |
|  | BJ323295 | Receptor protein kinase |
|  | DR731606 | DEAD-box ATP-dependent RNA helicase 42 |
|  | CK154549 | Site-specific DNA-methyltransferase |
|  | TC420083 | Sugar transporter protein |
| TamiR50 | DR735117 | Peptidase C14, caspase catalytic subunit p20 precursor |
|  | GH728249 | CCT motif family protein, expressed |
|  | CK156857 | Beta3-glucuronyltransferase |
|  | BQ608454 | Chromosome segregation ATPases-like protein |
| TamiR51 | TC372128 | RuBisCO large subunit-binding protein subunit alpha, chloroplast precursor |
|  | DR735926 | E3 ubiquitin-protein ligase Hakai1 |
|  | TC386992 | Sec14 like protein |
|  | CK200194 | Dehydrin |
| TamiR52 | DR735657 | WW domain binding protein 11 pseudogene 1 (WBP11P1) on chromosome 18 |
|  | TC376047 | Ribulose bisphosphate carboxylase small chain PWS4.3 |
|  | CK159515 | Peptidyl-prolyl cis-trans isomerase; n=1; Dictyostelium discoideum |
|  | DR732356 | Tryptophanyl-tRNA synthetase |
|  | DR732607 | 23S rRNA (uracil-5-)-methyltransferase |
